# Preclinical pharmacological profiles of cofrogliptin, a novel and bi-weekly DPP-4 inhibitor

**DOI:** 10.3389/fphar.2025.1702101

**Published:** 2026-01-05

**Authors:** Xiaoli Gou, Caixia Dou, Pingming Tang, Xiaoxiao Zheng, Chen Zhang, Jianmin Wang, Qingyuan Meng, Ju Wang

**Affiliations:** Haisco Pharmaceutical Group Co., Ltd., Chengdu, Sichuan, China

**Keywords:** bi-weekly, cofrogliptin, dipeptidyl peptidase 4, DPP-4 inhibitor, HSK7653, long-acting, type 2 diabetes

## Abstract

**Objective:**

DPP-4 inhibitors are now established agents for glycaemic control in diabetes. Herein, this study systematically characterized cofrogliptin and elucidated its pharmacology, pharmacokinetics, and therapeutic efficacy for type 2 diabetes.

**Methods:**

*In vitro* pharmacological characterization of cofrogliptin was performed using recombinant enzyme inhibition assays, serum/plasma DPP-4 inhibitory activity profiling, and the SafetyScreen panel. *In vivo* DPP-4 inhibitory potency was evaluated via measurement of serum DPP-4 activity in ICR and *ob/ob* mice following a single oral administration of cofrogliptin. Additionally, the oral glucose tolerance test was conducted in ICR and *db/db* mice pre-treated with a single oral dose of cofrogliptin. For assessment of chronic therapeutic efficacy, *ob/ob* mice were used with intermittent dosing to simulate prolonged diabetes management. Finally, a translational pharmacokinetic-pharmacodynamic relationship was established across rats, dogs, and monkeys to elucidate the mechanistic basis of cofrogliptin’s long-acting therapeutic benefits.

**Results:**

The findings have revealed that cofrogliptin is a potent and selective DPP-4 inhibitor with an IC_50_ of 10.80 nM in the recombinant DPP-4 enzyme assay. In *ob/ob* mice, it exerted favorable anti-diabetic effects, superior to those of MK3102. Furthermore, cofrogliptin exhibited a robust PK-PD relationship across rats, dogs, and monkeys. Notably, following a single oral dose of 10 mg/kg cofrogliptin, monkeys exhibited sustained DPP-4 inhibition of approximately 80% through day 14. Specifically, cofrogliptin exhibited superior pharmacokinetic profiles compared to MK3102 in rodents, whereas its PK characteristics in non-rodents were comparable to those of MK3102. Based on allometric scaling analyses, cofrogliptin is predicted to display superior human pharmacokinetic properties relative to MK3102, with anticipated longer-acting features that align with its clinical dosing frequency.

**Conclusion:**

Consequently, as the only approved bi-weekly DPP-4 inhibitor, cofrogliptin is well-positioned to substantially improve medication adherence among patients with type 2 diabetes, thereby improving therapeutic outcomes.

## Graphical Abstract

**Figure:**
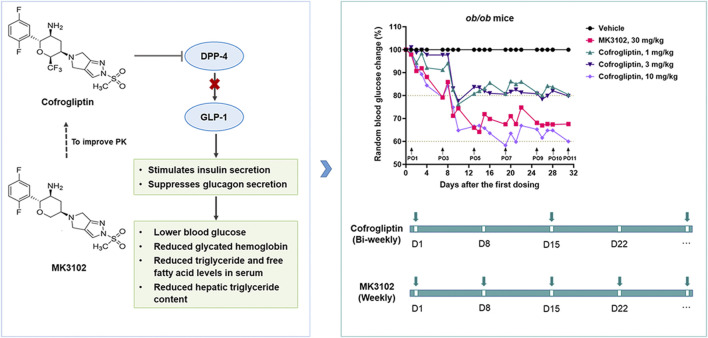

## 1 Introduction

Type 2 diabetes mellitus (T2DM), a chronic and progressive disease with complex pathophysiology, has witnessed an alarming upsurge in its prevalence rates across the globe, posing significant challenges to public health and healthcare systems (Tinajero and Malik, 2021; Abel et al., 2023; Yan et al., 2022). It is characterized by fasting hyperglycemia, impaired glucose tolerability and insulin resistance during the course of the disease. The chronically elevated blood glucose levels can give rise to severe systemic damage to multiple organs such as the heart, eyes, kidneys, blood vessels and nerves. Conventional antidiabetic drugs, including insulin, biguanides, sulfonylureas and thiazolidinediones, have effectively improved glucose control of diabetic patients. However, the clinical use of these drugs is typically accompanied with several disadvantages, such as the absence of oral availability, short half-lives, and unwanted side effects. Meanwhile, these drugs offer no obvious benefits for the long-term complications of diabetes (Davies et al., 2018; Yeh et al., 2021; American Diabetes Association Professional Practice Committee, 2024). In recent years, a new class of antidiabetic drugs has been introduced to treat T2DM, namely, dipeptidyl peptidase 4 (DPP-4) inhibitors, sodium–glucose cotransporter 2 (SGLT2) inhibitors, and glucagon-like peptide-1 (GLP-1) receptor agonists (Davies et al., 2018).

Incretin hormones (incretins) are a group of gastrointestinal peptides, such as GLP-1 and glucose-dependent insulinotropic polypeptide (GIP) (Nauck and Meier, 2016; Ansari et al., 2024). GLP-1 and GIP are released by specialized enteroendocrine L cells in the distal intestine and K cells in the proximal intestine, respectively, in response to nutrient ingestion. These hormones function through their respective receptors on pancreatic islet beta cells, thereby triggering the so-called incretin effect, which enhances insulin secretion in response to ingested glucose rather than intravenous glucose (Kazakos, 2011). However, the incretin effect is uniformly impaired in patients with T2DM. Therefore, enhancing incretin action has become a novel therapeutic approach to the treatment of T2DM (Nauck and Meier, 2016).

Dipeptidyl peptidase 4 (DPP-4), also known as CD26, belongs to a subfamily of the serine peptidase family S9 (Hua et al., 2023). It specifically targets dipeptides at the N-terminus of proline-containing peptides and is present in both membrane-bound and soluble forms (Hussain et al., 2019). The membrane-bound DPP-4 is predominantly located on the surfaces of endothelial cells (Palmieri and Ward, 1989) and epithelial cells (Yang et al., 2007), as well as on immune cells (Reinhold et al., 2002; Ohnuma et al., 2008). The highest expression of DPP-4 in humans is observed on the epithelial cells in the kidney, as well as in the small and large intestine (Shi et al., 2016). Additionally, it is present on the surfaces of hepatocytes, lung cells, and other cell types (Itou, 2013; Zou et al., 2020). The soluble DPP-4 circulates within the plasma and has high accessibility to the gut, liver, lung, and kidney. The extensive presence of DPP-4 across different tissues underpins its diverse biological activities. With its protease activity, it has been demonstrated that DPP-4 can act on over 30 peptide substrates, including GLP-1, GIP, pituitary adenylate cyclase-activating polypeptide (PACAP), high mobility group protein 1 (HMGB1) and several others (Mulvihill and Drucker, 2014; Huang et al., 2022).

Under physiological and pathological conditions, active GLP-1 and GIP are quickly degraded in the bloodstream by DPP-4 with a half-life of 1-7 min, thereby losing their insulinotropic effect (Mentlein et al., 1993; Gilbert and Pratley, 2020). Accordingly, DPP-4 represents a promising target for the treatment of diabetes. By inhibiting its protease activity, DPP-4 inhibitors lead to an elevation in the level of active GLP-1 and GIP. Thereby, they enhance insulin secretion and contribute to the maintenance of glucose homeostasis. To date, a number of DPP-4 inhibitors have been documented, including alogliptin (Feng et al., 2007), BI 1356 (linagliptin) (Thomas et al., 2008), sitagliptin (Mu et al., 2009), teneligliptin (Yoshida et al., 2012), DA-1229 (evogliptin) (Kim et al., 2012), PKF-275-055 (vildagliptin) (Akarte et al., 2012), MK3102 (omarigliptin) (Biftu et al., 2014), gemigliptin (Kim et al., 2016), trelagliptin (Grimshaw et al., 2016), DBPR108 (prusogliptin) (Yeh et al., 2021), ZD-2 (Fan et al., 2022), and so on. The majority of these DPP-4 inhibitors need to be administered twice or once a day because of their short half-lives. However, several studies have demonstrated that a considerable proportion of patients with diabetes exhibit poor medication adherence (Polonsky and Henry, 2016; Ekenberg et al., 2024). Importantly, poor medication adherence in T2DM is linked to inadequate glycemic control, higher morbidity and mortality rates, as well as increased costs for hospitalization and management of diabetes complications. Hence, there is a need for the development of innovative, safe and long-lasting DPP-4 inhibitors. On the other hand, although trelagliptin and omarigliptin, which have a once-weekly dosing regimen, have been introduced and significantly improved patient compliance, they still have obvious drawbacks. Studies have indicated that trelagliptin should be administered with caution in patients with renal dysfunction (Kaku, 2015). Moreover, omarigliptin may carry a higher risk of inducing serious cardiovascular issues, hepatobiliary adverse events, and prostate cancer when compared with other hypoglycemic medications (Stoimenis et al., 2017).

**FIGURE 1:**
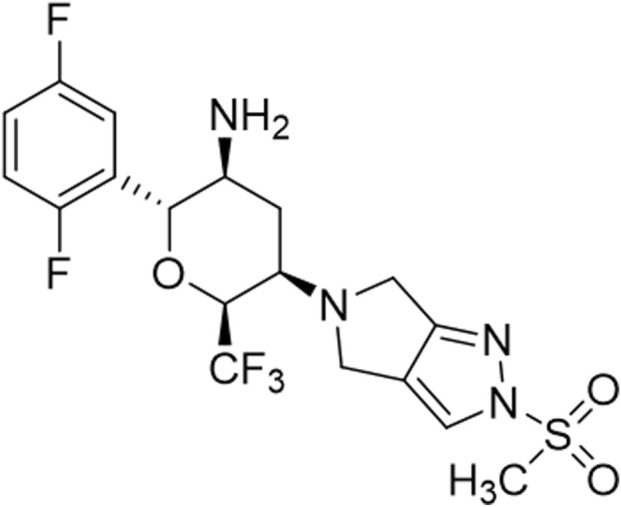
Chemical structure of cofrogliptin (HSK7653).

In an effort to design a novel, safe and exceptionally long-acting hypoglycemic medication, we have developed a DPP-4 inhibitor, cofrogliptin (also known as HSK7653, Figure 1), which requires administration only once every 2 weeks (Zhang et al., 2020). The purpose of the present study is to characterize the pharmacological profiles of cofrogliptin. The characterization focused on the following key aspects: (1) *in vitro* potency and selectivity; (2) *in vivo* inhibitory activity on DPP-4 activity in ICR and *ob/ob* mice; (3) oral glucose tolerance test (OGTT) in ICR mice; (4) OGTT in *db/db* mice; (5) long-term antidiabetic efficacy in o*b/ob* mice; (6) pharmacokinetic-pharmacodynamic (PK-PD) relationship in rats and dogs; (7) PK-PD relationship in monkeys.

## 2 Materials and methods

### 2.1 Ethical considerations

All animal studies were conducted in strict accordance with the Act on Welfare and Management of Animals in China and adhered to the National Institutes of Health (NIH) Guide for the Care and Use of Laboratory Animals. The protocols were meticulously reviewed and approved by the Institutional Animal Care and Use Committee (IACUC) of Shanghai Institute of Materia Medica, Chinese Academy of Sciences (Shanghai, China), Soochow University (Suzhou, China), 3D BioOptima (Suzhou) Co., Ltd (China), or Sichuan Plamei Biotechnology Co., Ltd. (Chengdu, China).

### 2.2 Animals

In the present study, C57BL/6J mice, Institute of Cancer Research (ICR) mice, B6.V-Lepob/J (*ob/ob*) mice, wild-type B6 mice, B6.Cg-m +/+ Leprdb/J (*db*/*db*) mice, Sprague-Dawley (SD) rats, beagle dogs, and rhesus monkeys were utilized. Details regarding the animals’ source and housing conditions are provided in the Supplementary Material. All animals were housed in a temperature- and humidity-controlled facility under a standard 12-h light/12-h dark cycle, with *ad libitum* access to food and water.

### 2.3 Reagents and drugs

Recombinant human DPP-4, DPP-8, and DPP-9 enzymes were purchased from Enzo Life Sciences (Farmingdale, NY, USA). Gly-pro-7-amido-4-methylcoumarin hydrobromide (Gly-Pro-7-AMC), a synthetic peptide, was obtained from Sigma-Aldrich (St. Louis, MO, USA) and used as a substrate to evaluate the activities of these enzymes. Glucose was also obtained from Sigma-Aldrich and dissolved in double-distilled water prior to use. The Accu-Chek Performa test strips, Tina-quant® HbA1c and fructosamine (FRA) assay kits were provided by Roche Diagnostics GmbH (Germany). The glucose assay kit and the ultra-sensitive mouse insulin ELISA kit were obtained from Shanghai Rongsheng Biotech Co., Ltd (China) and Crystal Chem Inc. (IL, USA), respectively, and were used to measure serum glucose levels and insulin concentrations. Triglycerides (TG), total protein (TP) and albumin (ALB) assay kits were purchased from Maccura Biotechnology Co., Ltd (Chengdu, China). Total cholesterol (TC) assay kit (Quick Auto Neo T-CHO) was obtained from Shino-Test Corporation (Japan). LabAssay™ HDL-Cholesterol (HDL-C), LabAssay™ LDL-Cholesterol (LDL-C) and LabAssay™ NEFA (FFA) assay kits were from FUJIFILM Wako Pure Chemical Industries, Ltd. (Japan).

Cofrogliptin and MK3102 were synthesized by Sichuan Haisco Pharmaceutical Co., Ltd (Chengdu, China). For *in vivo* studies, MK3102 was formulated into preparations containing 1% hydroxypropyl methylcellulose (HPMC), with the active ingredients at specified concentrations. Cofrogliptin was prepared in one of the following formulations: 1% HPMC, 0.5% sodium carboxymethyl cellulose (CMC-Na) exclusively for the PK-PD relationship study, or a mixture of 5% N,N-dimethylacetamide, 5% Solutol HS-15, and 90% saline for intravenous infusion.

### 2.4 *In vitro* enzyme inhibition assay

#### 2.4.1 Recombinant DPP-4 enzyme inhibition assay

An *in vitro* recombinant DPP-4 enzyme inhibition assay was carried out to evaluate the inhibitory effects of cofrogliptin and MK3102 on DPP-4 activity. In this assay, 10 µL of working solution of either cofrogliptin or MK3102 (0.128–10,000 nM), 10 µL of recombinant human DPP-4 (0.025 mU/μL), and 30 µL of assay buffer were mixed thoroughly. Subsequently, the reaction was initiated by adding 50 μL of assay buffer containing 10 µM Gly-Pro-7-AMC. Once Gly-Pro-7-AMC was introduced, the fluorescence intensity (FI) was promptly measured over a span of 10 min at a frequency of one detection per minute using an automated fluorescent plate reader (excitation 380 nm/emission 460 nm, FlexStation III, Molecular Devices, CA, USA).

#### 2.4.2 Endogenous DPP-4 inhibition assays

The inhibition of endogenous DPP-4 present in serum (male C57BL/6J mice and SD rats) or plasma (male beagle dogs and rhesus monkeys) samples was also evaluated following a similar assay protocol. In this case, 5 μL of serum or plasma served as the enzyme source. Subsequently, 10 µL of working solution of either cofrogliptin or MK3102 (0.128–10,000 nM) and 35 µL of assay buffer were incorporated into the reaction mixture. The reaction was initiated by adding 50 µL of 10 µM Gly-Pro-7-AMC. The fluorescence intensity (FI) was then monitored over an 18-min period, with readings taken every 3 min, using an automated fluorescent plate reader (excitation 380 nm/emission 460 nm, FlexStation III, Molecular Devices, CA, USA).

#### 2.4.3 Recombinant DPP-8 and DPP-9 enzyme inhibition assays

To assess the inhibition of DPP-8 and DPP-9, 10 µL of working solution of either cofrogliptin or MK3102 (0.0128–1,000 µM) was mixed with 5 µL of recombinant human DPP-8 or DPP-9 (2 ng/μL) and 35 µL of assay buffer. Then, the reaction was initiated by adding 50 µL of assay buffer containing Gly-Pro-7-AMC. Specifically, the concentration of Gly-Pro-7-AMC was set at 100 µM for DPP-8 and 20 µM for DPP-9. Finally, the fluorescence intensity (FI) was continuously monitored over a 30-min period, with measurements taken every 3 min using an automated fluorescent plate reader (excitation 380 nm/emission 460 nm, FlexStation III, Molecular Devices, CA, USA).

#### 2.4.4 Inhibition rate calculation

Based on the fluorescence intensity measurement results, a time-fluorescence intensity curve was constructed. The slope of the curve was calculated and used as the activity value. The percentage of inhibition was calculated using the formula: % Inhibition = [1 – (A_compound_ – A_blank_)/(A_negative control_ – A_blank_)] × 100%. Here, A_compound_ represents the activity value of the treated sample, while A_negative control_ and A_blank_ denote the activity values of the negative control and blank samples, respectively (Kim et al., 2005). In the negative control group, the working solution of either cofrogliptin or MK3102 was replaced by assay buffer. In the blank group, both the working solution and the enzymes were replaced by assay buffer.

### 2.5 Effects on DPP-4 activity in ICR and *ob/ob* mice

After an acclimation period, sixty male ICR mice (weighing 28–33 g) were evenly assigned to six treatment groups based on their body weights, and baseline serum samples were collected. The mice were then orally administered a single dose of cofrogliptin (1, 3, 10, or 30 mg/kg) or MK3102 (10 mg/kg). Mice in the vehicle-treated group received 1% HPMC. Serum samples were subsequently collected at 4, 10, 24, 34, 48, 58, 72, 82, and 96 h post-dosing for measurement of DPP-4 activity.

For DPP-4 inhibition assays, 60 B6.V-Lepob/J (*ob/ob*) mice (39–55 g, half male and half female) and 10 wild-type B6 mice (15–25 g, half male and half female) were used. The *ob/ob* mice were evenly assigned to six treatment groups based on their body weights, and baseline serum samples were collected. The mice were orally treated with a single dose of cofrogliptin (1, 3, 10, or 30 mg/kg) or MK3102 (30 mg/kg). Animals in the wild-type mice group and vehicle-treated *ob/ob* mice group received 1% HPMC. Serum samples were collected at 4, 10, 24, 34, 48, 58, 72, 82, and 96 h post-dosing for measurement of DPP-4 activity.

DPP-4 activity was measured using a method adapted from a published protocol (Villhauer et al., 2003). Briefly, 5 μL of serum sample was mixed with 45 μL of assay buffer and pre-incubated at room temperature for 5 min. Subsequently, 10 μL of Gly-Pro-7-AMC (0.1 mM) and 40 μL of assay buffer were added. Fluorescence intensity (FI) was monitored as previously described in the endogenous DPP-4 inhibition assays. Based on the measurement results, a time-fluorescence intensity curve was plotted after subtracting the blank background. The slope of the curve was taken as the activity value. The serum DPP-4 activity before dosing was set as 100%. The relative activities of serum DPP-4 at each time point after dosing were calculated using the following formula: DPP-4 activity (%) = (Activity after dosing/Activity before dosing) × 100%.

### 2.6 Oral glucose tolerance test (OGTT) in ICR mice

Following an acclimation period, 100 male ICR mice (weighing 27–31 g) were randomly assigned into two parallel groups based on body weights. Each parallel group was further divided into five subgroups: the vehicle group, the 1 mg/kg cofrogliptin group, the 3 mg/kg cofrogliptin group, the 10 mg/kg cofrogliptin group, and the 10 mg/kg MK3102 group. In the first parallel group, mice were orally administered either 1% HPMC (vehicle) or different doses of cofrogliptin or MK3102, and subsequently underwent OGTT at 24 h and 72 h post-treatment. In the second parallel group, mice received the same treatments and underwent OGTT at 48 h and 96 h post-treatment. Prior to the OGTT, all animals were fasted overnight and then orally administered 2.5 g/kg glucose. Blood glucose levels were measured using the Accu-Chek Performa test strips at baseline (before glucose administration) and at 0.25, 0.5, 1, and 2 h after glucose administration. The area under glucose increment level-time curve within 2 h (AUC_Glu, incre_) was calculated using the trapezoidal rule with the following formula: AUC_Glu, incre_ = [(BG_0h_ + BG_0.25h_) × 0.25 + (BG_0.25h_ + BG_0.5h_) × 0.25 + (BG_0.5h_ + BG_1h_) × 0.5 + (BG_1h_ + BG_2h_)] × 0.5, where BG_0h_, BG_0.25h_, BG_0.5h_, BG_1h_, and BG_2h_ represent the blood glucose increment levels at 0 (baseline), 0.25, 0.5, 1, and 2 h after glucose loading, respectively. The percentage decrease in AUC_Glu, incre_ was computed using the following formula (AUC_Glu, incre_vehicle_ – AUC_Glu, incre_drug_)/AUC_Glu, incre_vehicle_ × 100%, where AUC_Glu, incre_vehicle_ and AUC_Glu, incre_drug_ are the AUC_Glu, incre_ values in the vehicle group and the drug-treated group, respectively.

### 2.7 Oral glucose tolerance test (OGTT) in *db/db* mice

Following weaning, 157 B6.Cg-m +/+ Leprdb/J (*db*/*db*) mice (31–46 g, 85 males and 72 females) were individually housed and maintained on a high-fat diet. At 7 weeks of age, the mice underwent a 10-h fasting period, after which fasting blood glucose and serum insulin levels were measured. Based on these metabolic parameters (fasting blood glucose, serum insulin) combined with body weight measurements, 70 *db*/*db* mice were systematically stratified into seven experimental groups (*n* = 10 per group, half male and half female) with balanced metabolic profiles (Supplementary Table 1). In the vehicle-treated group, mice were orally administered 1% HPMC 48 h before glucose administration. In the drug-treated groups, mice received oral doses of cofrogliptin at 10 mg/kg, administered 10, 24, 34, 48, 58, or 72 h prior to glucose administration. On the day of the oral glucose tolerance test, the mice were fasted before glucose administration, and the test was conducted 10 h later. The mice were then orally administered glucose at a dose of 2.5 g/kg. Blood samples were collected before glucose administration and at 0.25, 0.5, 1, and 2 h post-administration. Then, serum samples were obtained to measure glucose levels, insulin concentrations, and DPP-4 activity, employing the methods elaborated in the Supplementary Material and the section titled “Effects on DPP-4 activity in ICR and *ob/ob* mice.” Subsequently, the AUC_Glu_ and percentage decrease in AUC_Glu_ were determined using the aforementioned method. Similarly, the area under insulin concentration-time curve within 2 h (AUC_Ins_) was also calculated using the trapezoidal rule. The percentage increase in AUC_Ins_ was computed using the following formula (AUC_Ins, drug_–AUC_Ins, vehicle_)/AUC_Ins, vehicle_ × 100%, where AUC_Ins, drug_ and AUC_Ins, vehicle_ are the AUC_Ins_ values in the drug-treated group and the vehicle group, respectively.

### 2.8 Long-term antidiabetic efficacy evaluation of cofrogliptin in *ob/ob* mice

After weaning, 238 B6.V-Lepob/J (*ob/ob*) mice (30–49 g, 129 males and 109 females) and 22 wild-type B6 mice (15–22 g, 10 males and 12 females) were individually housed. The *ob/ob* mice were given a high-fat diet, whereas the wild-type mice were maintained on a standard diet. At 7 weeks of age, *ob/ob* mice underwent measurements of random blood glucose, fasting blood glucose, postprandial body weight, fasting body weight, glycated hemoglobin (HbA1c), and serum insulin concentrations. Based on these parameters, 60 *ob/ob* mice were allocated into five groups, with 12 mice per group (half male and half female). Additionally, a separate cohort of wild-type mice was designated as the normal control group, consisting of six males and six females. The detailed grouping information and baseline parameters of the animals prior to drug administration are thoroughly documented in Supplementary Table 2. After the initial grouping, mice in each experimental group were orally administered the test drug (MK3102 or cofrogliptin) at an interval of once every 3 days. Simultaneously, mice in the wild-type control group and the vehicle control group received 1% HPMC at the same frequency. The experimental timeline was defined with the first day of drug administration designated as Day 1. Drug dosing continued for a total of 11 administrations. The experiment was terminated on Day 31, which coincided with the 11th drug administration. The experimental schedule is provided in detail in Supplementary Table 3.

#### 2.8.1 Random and fasting blood glucose measurement

During the dosing period, random and fasting blood glucose levels were measured in the 1st, 3rd, 5th, 7th, 9th, 10th, and 11th dosing cycles using the Accu-Chek Performa test strips. Random blood glucose was assessed between 8:00 and 9:00 a.m. Fasting blood glucose was measured after a 6-h fasting period (with free access to water), specifically between 14:00 and 15:00. From the 1st to the 9th dosing cycles, random blood glucose levels were measured at four time points: before dosing (72 h after the last dose) and at 24, 48, and 72 h post-dosing. Fasting blood glucose levels were measured at three time points: 6, 30, and 54 h post-dosing. In the 10th dosing cycle, only fasting blood glucose levels were recorded on the dosing day. In the 11th dosing cycle, random and fasting blood glucose levels were measured only on the day of dosing. The percentage change in blood glucose was calculated using the following formula: blood glucose change (%) = (1–BG_drug_/BG_vehicle_) × 100%, where BG_drug_ represents the blood glucose level in the drug-treated group, and BG_vehicle_ denotes the blood glucose level in the vehicle group.

#### 2.8.2 Postprandial and fasting body weight recording

During the treatment period, the body weights of the mice were measured daily, and their fasting body weights were recorded concurrently with the fasting blood glucose measurements.

#### 2.8.3 Glycated hemoglobin (HbA1c) and insulin analysis

After the 10th administration (on Day 28), two blood samples were collected from each mouse after a 6-h fasting period. One of the samples was immediately placed into Eppendorf tubes treated with an anticoagulant. The other blood sample was used for serum separation. Subsequently, the HbA1c levels in whole blood were measured using the Tina-quant® HbA1c assay kit in conjunction with an automatic biochemical analyzer (HITACHI 7020, Japan) to ensure precise quantification (Liu et al., 2008). Insulin concentrations in serum were analyzed employing the methods elaborated in the Supplementary Material.

#### 2.8.4 Food consumption and water intake assessment

Food consumption and water intake of mice were assessed at specified time points (Supplementary Table 3).

#### 2.8.5 Animal dissection

On Day 31, following a 6-h fasting period, blood samples were collected from the mice to separate the serum. This serum was subsequently subjected to analysis using an automatic biochemical analyzer (HITACHI 7020, Japan) for a comprehensive biochemical analysis, which included the determination of fructosamine (FRA), triglycerides (TG), total cholesterol (TC), high-density lipoprotein cholesterol (HDL-C), low-density lipoprotein cholesterol (LDL-C), total protein (TP), and albumin (ALB) levels. The biochemical analysis was performed using corresponding assay kits, which are detailed in the “Reagents and drugs” section. Additionally, the serum was analyzed for free fatty acids (FFA) using the LabAssay™ NEFA (FFA) assay kit. The animals then underwent dissection. Various tissues, including the liver, kidneys, spleen, pancreas, heart, and multiple fat depots (epididymal, mesenteric, scapular, subcutaneous, perirenal, and inguinal fats), were meticulously isolated and weighed. The ratios of these tissue weights to body weight were calculated to assess tissue mass relative to overall body weight. Additionally, the pancreases were harvested from mice in the wild-type control, vehicle control, 10 mg/kg cofrogliptin, and 30 mg/kg MK3102 groups. Subsequently, they were fixed and embedded in paraffin. This step was done to prepare for subsequent analysis of insulin and glucagon expression via immunofluorescence staining. Moreover, hepatic triglyceride content was determined. The detailed protocols for immunofluorescence staining to analyze insulin and glucagon expression in pancreatic tissue, as well as the methods for determining hepatic triglyceride content, are provided in the Supplementary Material.

### 2.9 PK-PD relationship of cofrogliptin in inhibiting plasma DPP-4 activity in rats and dogs

Eighteen SD rats (192–260 g, half male and half female) were randomly divided into three experimental groups, with each group consisting of six rats (three males and three females). Then, the rats were administered cofrogliptin orally at different doses (3, 10, and 30 mg/kg). Blood samples were collected pre-dose and at various post-dose time points, including 30 min, 1, 2, 4, 8, 24, 48, 72, 120, and 168 h.

Eighteen beagle dogs (5.6–6.6 kg, half male and half female) were also used in the study and were divided into three groups, each containing six dogs (three males and three females). Subsequently, the dogs received a single oral administration of cofrogliptin at the specified doses (1, 3, or 10 mg/kg). Blood samples were collected before dosing and at 15 min, 30 min, 1, 2, 4, 8, 12, 24, 36, 48, 72, 96, 120, 144, and 168 h post-dose.

At each sampling time point, a specified volume of whole blood was collected from the jugular vein. Specifically, 0.2 mL was drawn from each rat, while 0.6 mL was collected from each dog. Immediately following the collection, the blood samples were transferred into tubes containing EDTA-K_2_, an anticoagulant. The samples were then centrifuged at 5,500 rpm for 10 min to separate the plasma. For each sample, 30–50 μL of the separated plasma was aliquoted into a labeled microcentrifuge tube and stored at ≤ −70 °C in an ultra-low temperature freezer for subsequent DPP-4 enzyme activity assays, which were conducted in accordance with the method detailed in the “Effects on DPP-4 activity in ICR and *ob/ob* mice” section. The remaining plasma was transferred to a separate labeled microcentrifuge tube and stored at ≤ −20 °C for subsequent determination of drug concentrations using validated analytical methods (Supplementary Material).

### 2.10 PK-PD relationship of cofrogliptin in inhibiting plasma DPP-4 activity in monkeys

Four male rhesus monkeys were randomly assigned to two experimental groups, with two monkeys in each group. The positive control group received 10 mg/kg of MK3102 via oral gavage, while the test group received 10 mg/kg of cofrogliptin through the same route of administration. Blood samples were collected at a total of 34 time points: pre-dose (0 h) and at 33 post-dose time points, specifically at 0.5, 1, 2, 4, 8, 12, 24, 32, 48, 56, 72, 80, 96, 104, 120, 128, 144, 152, 168, 192, 216, 240, 264, 288, 312, 336, 360, 384, 408, 432, 456, 480 and 504 h. For each sampling, 0.6 mL of blood was drawn from the forelimb vein into EDTA-Na_2_ anticoagulant tubes, which were then gently inverted several times to ensure thorough mixing. The blood samples were subsequently centrifuged at 5,500 rpm for 10 min to separate the plasma. Precisely 50 μL of the separated plasma was transferred to microcentrifuge tubes, while the remaining plasma was aliquoted into separate microcentrifuge tubes. All plasma samples were immediately stored in an ultra-low temperature freezer at −80 °C until further biochemical and pharmacokinetic analyses were conducted. Subsequently, DPP-4 enzyme activity assay and drug concentration analysis were performed using validated methods (Supplementary Material).

### 2.11 Statistical analysis

All study endpoints are presented as the mean ± standard error of the mean (SEM). Statistical analyses were conducted using GraphPad Prism software (San Diego, CA, USA). To determine significant differences among treatment groups, one-way analysis of variance (ANOVA) was applied, followed by *post hoc* testing using Dunnett’s multiple comparison test. For pairwise comparative analyses, a two-tailed unpaired Student’s t-test was employed to evaluate statistically significant differences between individual groups. A *p*-value less than 0.05 was considered statistically significant.

## 3 Results

### 3.1 *In vitro* potency and selectivity of cofrogliptin

**FIGURE 2:**
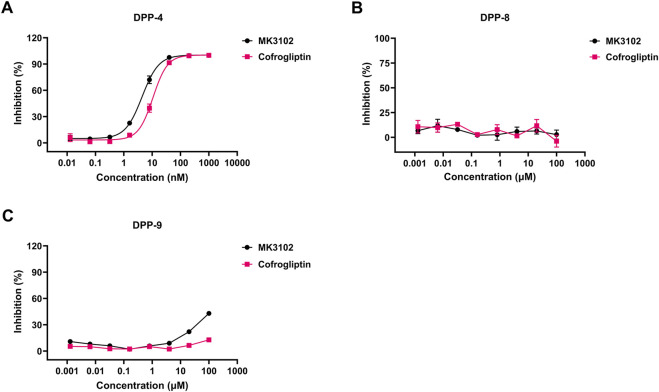
*In vitro* potency and selectivity of cofrogliptin in recombinant enzyme inhibition assays. Inhibitory activity of cofrogliptin on recombinant human DPP-4 **(A)**, DPP-8 **(B)**, and DPP-9 **(C)**. Data are expressed as mean ± SEM. *n* = 3 per group.

Cofrogliptin exhibited potent inhibitory activity against recombinant human DPP-4 with a half-maximal inhibitory concentration (IC_50_) of 10.80 ± 1.83 nM. The corresponding IC_50_ for MK3102 was 4.45 ± 0.82 nM, approximately half of that of cofrogliptin (Figure 2). Cofrogliptin also potently inhibited DPP-4 activity in serum or plasma across all tested species, including mice, rats, dogs, and monkeys, with IC_50_ values varying by only 1.32-fold across species (Supplementary Figure 1). Notably, cofrogliptin showed remarkable selectivity for DPP-4 over the closely related enzymes DPP-8 and DPP-9, with IC_50_ values exceeding 100 μM (Figure 2). This pharmacological profile translates to >9,000-fold selectivity for recombinant DPP-4 relative to DPP-8 and DPP-9. Additionally, at a concentration of 10 μM, cofrogliptin demonstrated no significant activity against an extensive panel of 127 targets, including receptors, ion channels, transporters, and enzymes (Supplementary Table 4). These findings further underscore the highly favorable target specificity profile of cofrogliptin.

### 3.2 *In vivo* inhibitory activity of cofrogliptin on DPP-4 activity in ICR and *ob/ob* mice

**FIGURE 3:**
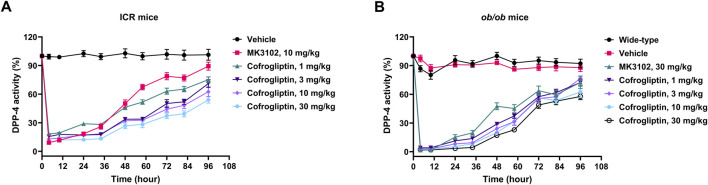
*In vivo* inhibitory activity of cofrogliptin on serum DPP-4 activity in ICR **(A)** and *ob/ob* mice **(B)**. Data are expressed as mean ± SEM. *n* = 10 per group.

The *in vivo* inhibitory activity of cofrogliptin against serum DPP-4 was further investigated in ICR and *ob/ob* mice. After a single oral administration of different doses (1–30 mg/kg) of cofrogliptin to ICR mice, the serum DPP-4 activity was significantly inhibited within 96 h, and the serum DPP-4 activity decreased in a dose-dependent manner (Figure 3). Upon administration of cofrogliptin at 1 mg/kg, serum DPP-4 activity was significantly reduced, with an inhibition rate exceeding 70% for up to 34 h. When the dose of cofrogliptin was escalated to 3 and 10 mg/kg, the inhibitory effect on DPP-4 was even more pronounced, achieving over 80% inhibition within the first 34 h and maintaining above 60% up to 58 h. At the highest dose tested (30 mg/kg), cofrogliptin demonstrated a robust inhibitory profile, sustaining greater than 70% inhibition for 58 h and greater than 60% for 82 h. At the last time point (96 h), the inhibition rates for the 10 mg/kg and 30 mg/kg doses of cofrogliptin were 37.5% and 46.0%, respectively. In contrast, the positive control group receiving 10 mg/kg MK3102 exhibited greater than 70% DPP-4 inhibition within 34 h, but its efficacy waned over time, with inhibition rates falling to 32.6% at 58 h and further declining to 10.7% by 96 h.

In line with the results observed in ICR mice, a single oral administration of different doses of cofrogliptin to *ob/ob* mice elicited a significant and sustained inhibition of serum DPP-4 activity over a 96-h period (Figure 3). Notably, the lower doses of cofrogliptin (1, 3, and 10 mg/kg) achieved inhibition rates of serum DPP-4 activity exceeding 70% within the first 48 h, and these rates still remained above 60% until 58 h. The higher dose of cofrogliptin (30 mg/kg) showed robust inhibitory effects, maintaining inhibition rates of serum DPP-4 activity above 70% for up to 58 h and reaching an inhibition rate of 42.3% at the 96-h time point. For comparison, the positive control group treated with 30 mg/kg MK3102 showed a DPP-4 activity inhibition rate of 54.8% at 58 h, which then decreased to 27.6% by 96 h. Importantly, cofrogliptin’s inhibitory effect on serum DPP-4 activity in *ob/ob* mice did not show any significant sex-related differences (Supplementary Figure 2).

### 3.3 Reduction in glucose excursion during oral glucose tolerance test induced by cofrogliptin in ICR mice

In the oral glucose tolerance test (OGTT), oral administration of 2.5 g/kg glucose to ICR mice in the vehicle-treated control group remarkably increased blood glucose levels, which peaked at 30 min after glucose loading (Supplementary Figure 3). A single oral administration of cofrogliptin at doses of 1–10 mg/kg to ICR mice resulted in a significant reduction in the area under blood glucose increment level-time curve (AUC_Glu, incre_) after 24 h (Table 1). This reduction exhibited a robust dose-dependent trend, with the percentage decrease of AUC_Glu, incre_ ranging from 27.35% to 40.03% (Supplementary Table 5). The positive control group, treated with 10 mg/kg MK3102, also achieved a substantial AUC_Glu, incre_ reduction of 37.65%. There was no statistically significant difference in AUC_Glu, incre_ between the 10 mg/kg MK3102 group and the cofrogliptin-treated groups (*p* > 0.05). However, the AUC_Glu, incre_ in the 10 mg/kg MK3102 group was numerically higher than that in the 10 mg/kg cofrogliptin group. Forty-eight hours after dosing, the AUC_Glu, incre_ values in the cofrogliptin-treated groups (1–10 mg/kg) continued to be significantly lower than those in the vehicle-treated control group, with reduction rates ranging from 29.75% to 36.54%. The positive control group (10 mg/kg MK3102) also demonstrated a decrease in AUC_Glu, incre_ compared to the vehicle control group (*p* = 0.08), with a reduction rate of 21.18%. Similarly, while no statistically significant difference in AUC_Glu, incre_ was observed between the 10 mg/kg MK3102 group and the cofrogliptin-treated groups (*p* > 0.05), the AUC_Glu, incre_ in the 10 mg/kg MK3102 group was numerically higher than that in all cofrogliptin-treated groups. By 72 h, only the 10 mg/kg cofrogliptin group maintained a lower AUC_Glu, incre_ than the vehicle control group (*p* = 0.08), with an AUC_Glu, incre_ reduction rate of 20.95%. The other groups did not show significant differences from the vehicle-treated control group, with AUC_Glu, incre_ reduction rates below 20%. At 96 h post-dosing, no significant differences were observed between any of the treatment groups and the vehicle control group, with all AUC_Glu, incre_ reduction rates remaining below 20%.

**TABLE 1:** Effects of cofrogliptin on the area under blood glucose increment level-time curve (AUC_Glu, incre_) in ICR mice.

Group	AUC_Glu, incre_ (mmol/L × h)			
	24 h	48 h	72 h	96 h
Vehicle	14.74 ± 0.63	12.37 ± 0.63	9.07 ± 0.27	9.44 ± 0.61
MK3102, 10 mg/kg	9.19 ± 0.84***	9.75 ± 1.05 (*p* = 0.08)	7.95 ± 0.51	8.62 ± 0.70
Cofrogliptin, 1 mg/kg	10.71 ± 1.32**	8.69 ± 0.85**	7.82 ± 0.69	8.61 ± 0.93
Cofrogliptin, 3 mg/kg	9.89 ± 0.66***	7.93 ± 0.61***	7.68 ± 0.73	8.89 ± 0.91
Cofrogliptin, 10 mg/kg	8.84 ± 0.65***	7.85 ± 0.73***	7.17 ± 0.60 (*p* = 0.08)	8.84 ± 1.08

**p < 0.01, ***p < 0.001, vs. Vehicle, one-way ANOVA, followed by Dunnett’s *post hoc* test. Data are expressed as mean ± SEM. n = 10 per group.

Therefore, a single oral dose of cofrogliptin significantly lowered the increase in blood glucose levels following glucose loading in ICR mice, in a dose-dependent manner. The duration of its efficacy was also dose-related. The 1 and 3 mg/kg doses of cofrogliptin remained effective for up to 48 h, while the 10 mg/kg dose sustained its effect for up to 72 h. In contrast, the positive control (10 mg/kg MK3102) maintained efficacy for a maximum of 48 h post-dosing.

### 3.4 Effects of cofrogliptin on blood glucose, serum insulin levels and DPP-4 activity in *db/db* mice

In *db/db* mice, oral administration of 2.5 g/kg glucose elicited a marked elevation in blood glucose levels, with a peak observed at 30 min post-glucose loading (Supplementary Figure 4A). Pre-treatment with 10 mg/kg cofrogliptin at various time intervals prior to glucose loading effectively attenuated the glycemic response, as evidenced by a reduction in the area under blood glucose level-time curve (AUC_Glu_). Specifically, cofrogliptin administration 10–58 h before glucose loading decreased AUC_Glu_ by 12.21%–24.49%, with the most pronounced effect observed when cofrogliptin was administered 24 h in advance (Table 2).

**TABLE 2:** Effects of cofrogliptin on the area under blood glucose-time curve (AUC_Glu_) and percentage decrease in AUC_Glu_ in *db/db* mice.

Group		AUC_Glu_ (mmol/L × h)	Percentage decrease of AUC_Glu_ (%)
Vehicle	—	81.85 ± 3.25	—
Cofrogliptin, 10 mg/kg	10 h	63.56 ± 3.99**	22.35
Cofrogliptin, 10 mg/kg	24 h	61.81 ± 4.03**	24.49
Cofrogliptin, 10 mg/kg	34 h	67.57 ± 3.72*	17.45
Cofrogliptin, 10 mg/kg	48 h	66.74 ± 3.73*	18.46
Cofrogliptin, 10 mg/kg	58 h	67.13 ± 3.89*	17.98
Cofrogliptin, 10 mg/kg	72 h	71.85 ± 3.88	12.21

*p < 0.05, **p < 0.01, vs. Vehicle, one-way ANOVA, followed by Dunnett’s *post hoc* test. Data are expressed as mean ± SEM. n = 10 per group.

Regarding insulin dynamics, *db/db* mice exhibited a rapid increase in serum insulin levels following oral glucose administration, reaching a peak at 15 min post-glucose loading (Supplementary Figure 4B). Pretreatment with 10 mg/kg cofrogliptin at 10, 24, or 34 h before glucose loading significantly enhanced the area under serum insulin level-time curve (AUC_ins_), with increases ranging from 36.74% to 43.27%. Administration of cofrogliptin 48 h prior to glucose loading also resulted in a substantial 23.32% increase in AUC_ins_. However, pretreatment 58 or 72 h before glucose loading did not significantly impact serum insulin levels (Table 3).

**TABLE 3:** Effects of cofrogliptin on the area under insulin level-time curve (AUC_Ins_) and percentage increase in AUC_Ins_ in *db/db* mice.

Group		AUC_Ins_ (mmol/L × h)	Percentage increase of AUC_Ins_ (%)
Vehicle	—	23.41 ± 1.08	—
Cofrogliptin, 10 mg/kg	10 h	32.01 ± 2.01*	36.74
Cofrogliptin, 10 mg/kg	24 h	33.54 ± 2.88**	43.27
Cofrogliptin, 10 mg/kg	34 h	32.41 ± 2.57*	38.45
Cofrogliptin, 10 mg/kg	48 h	28.87 ± 2.40	23.32
Cofrogliptin, 10 mg/kg	58 h	23.67 ± 1.95	1.11
Cofrogliptin, 10 mg/kg	72 h	22.07 ± 1.54	−5.72

*p < 0.05, **p < 0.01, vs. Vehicle, one-way ANOVA, followed by Dunnett’s *post hoc* test. Data are expressed as mean ± SEM. n = 10 per group.

Additionally, a single oral dose of 10 mg/kg cofrogliptin in *db/db* mice effectively inhibited serum DPP-4 activity across multiple time points post-dosing, specifically at 10, 24, 34, 48, 58, and 72 h. The inhibitory effect on serum DPP-4 activity was most pronounced within 48 h post-dosing, with an inhibition rate exceeding 70%. This inhibition rate remained above 68% up to 58 h post-dosing (Supplementary Figure 5).

Collectively, a single oral dose of cofrogliptin significantly reduced blood glucose levels following glucose loading, increased glucose-induced serum insulin levels, and inhibited serum DPP-4 activity in *db/db* mice.

### 3.5 Long-term antidiabetic efficacy of cofrogliptin in *ob/ob* mice

As anticipated, *ob/ob* mice exhibited substantially higher levels across multiple parameters compared with normal wild-type mice. These parameters included random and fasting blood glucose, postprandial and fasting body weight, food consumption, water intake, glycated hemoglobin (HbA1c), fructosamine (FRA), serum insulin, and hepatic triglycerides. Additionally, *ob/ob* mice showed obvious abnormalities in serum lipid metabolism and impaired pancreatic islet function.

**FIGURE 4:**
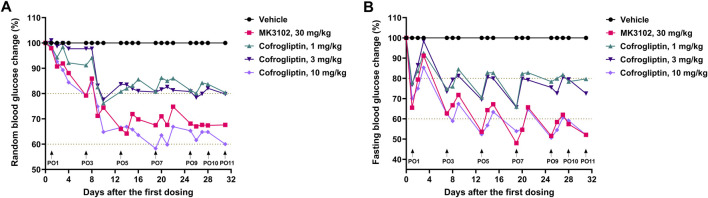
Effects of long-term oral administration of cofrogliptin on random **(A)** and fasting **(B)** blood glucose change in *ob/ob* mice. Data are expressed as mean ± SEM. *n* = 12 per group.

Compared to the vehicle-treated group (model control group), long-term oral administration of cofrogliptin (1–10 mg/kg) effectively reduced random and fasting blood glucose levels in *ob/ob* mice in a dose-dependent manner, achieving maximum reduction rates of 41.7% and 49.3% at the 10 mg/kg dose, respectively (Figure 4) (Zhang et al., 2020). In contrast, the 30 mg/kg MK3102 treatment achieved maximum reduction rates of 35.8% for random blood glucose and 52.0% for fasting blood glucose (Figure 4). To objectively compare the reduction in random and fasting blood glucose levels among different groups, we calculated the mean reduction rates throughout the experiment. For random blood glucose, the 1, 3, and 10 mg/kg cofrogliptin groups showed mean reduction rates of 14.0%, 13.8%, and 28.8%, respectively, whereas the 30 mg/kg MK3102-treated group exhibited a 25.2% reduction. In terms of fasting blood glucose, the same cofrogliptin doses yielded mean reduction rates of 20.3%, 21.7%, and 38.7%, respectively, compared to 36.9% in the 30 mg/kg MK3102-treated group.

Long-term oral administration of cofrogliptin also reduced glycated hemoglobin (HbA1c) and fructosamine (FRA) levels while increasing serum insulin concentrations (Supplementary Table 7). Furthermore, cofrogliptin treatment ameliorated the hyperlipidemic phenotype in *ob/ob* mice, as evidenced by significant reductions in key lipid parameters: triglyceride (TG) levels decreased by 9.3%–31.3%, free fatty acid (FFA) levels by 20.7%–31.0%, and hepatic triglyceride content by 12.2%–29.0% (Supplementary Tables 8, 9). Notably, although repeated administration of cofrogliptin once every 3 days did not significantly impact body weight, food intake, adipose tissue weight, or organ weights in *ob/ob* mice, it did lead to a reduction in water intake (Supplementary Figure 6, Supplementary Tables 10, 11).

To further evaluate the efficacy of cofrogliptin in *ob/ob* mice, immunofluorescence staining was conducted to examine insulin and glucagon expression in pancreatic tissue (Supplementary Figure 7). In the normal wild-type group, mouse islets were plump with abundant cytoplasm, showing abundant insulin-stained granules with strong staining intensity, while glucagon-stained granules were regularly arranged. In contrast, the islets of *ob/ob* mice in the vehicle-treated group exhibited atrophy, characterized by weak and irregularly distributed insulin-stained granules and disorganized glucagon-stained granules. However, in the 10 mg/kg cofrogliptin treatment group, islet contours were well-defined, and insulin-stained granules were significantly increased, featuring rich cytoplasm, enlarged staining areas, and enhanced intensity. Similar improvements were observed in the 30 mg/kg MK3102 positive control group.

The above results indicate that repeated administration of cofrogliptin exerted a favorable therapeutic effect on diabetes in *ob/ob* mice.

### 3.6 PK-PD relationship of cofrogliptin in inhibiting plasma DPP-4 activity in rats and dogs

**FIGURE 5:**
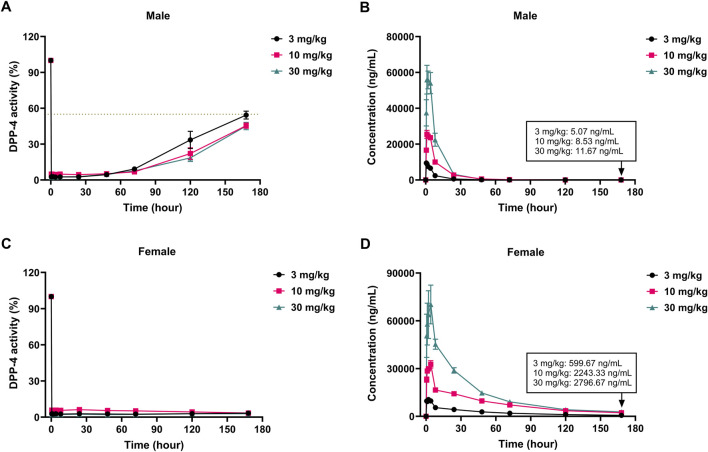
PK-PD relationship of cofrogliptin in inhibiting plasma DPP-4 activity in SD rats. *In vivo* inhibitory activity of cofrogliptin on plasma DPP-4 activity in male **(A)** and female **(C)** rats. Pharmacokinetic profiles of cofrogliptin in male **(B)** and female **(D)** rats. Data are expressed as mean ± SEM. *n* = 3 per group.

As illustrated in Figure 5, single oral administration of cofrogliptin at 3, 10, or 30 mg/kg to SD rats induced significant reductions in plasma DPP-4 activity. In male rats, plasma DPP-4 inhibition rates at 30 min post-administration were 97.4%, 95.1%, and 97.5%, corresponding to plasma cofrogliptin concentrations of 9,460, 16,613, and 37,460 ng/mL, respectively. At 72 h, inhibition rates remained above 90% (90.8%–93.2%) with concentrations ranging from 39.8 to 161 ng/mL. By 168 h, inhibition rates declined to 45.7%–54.9% alongside concentrations of 5.1–11.7 ng/mL. In female rats, 30-min inhibition rates were 97.3%, 94.2%, and 97.8% with respective concentrations of 9,611, 23,047, and 50,596 ng/mL. Plasma DPP-4 inhibition rates at 72 h (94.8%–97.7%) and 168 h (96.6%–97.2%) consistently exceeded 90%, paired with concentrations of 1,912–9,134 ng/mL and 600–2,797 ng/mL, respectively. Notably, female rats demonstrated significantly higher plasma drug concentrations and prolonged DPP-4 inhibition compared to males, indicative of sex-dependent pharmacokinetics. All dose groups exhibited rapid and potent DPP-4 inhibition, underscoring cofrogliptin’s efficacy profile in rats (Supplementary Figure 8).

**FIGURE 6:**
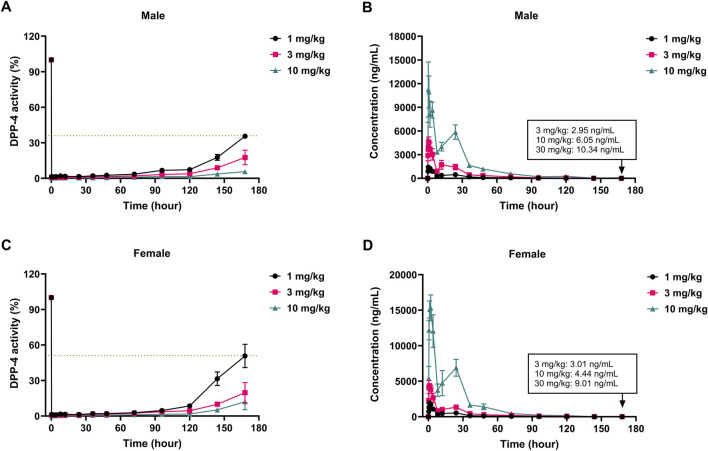
PK-PD relationship of cofrogliptin in inhibiting plasma DPP-4 activity in beagle dogs. *In vivo* inhibitory activity of cofrogliptin on plasma DPP-4 activity in male **(A)** and female **(C)** dogs. Pharmacokinetic profiles of cofrogliptin in male **(B)** and female **(D)** dogs. Data are expressed as mean ± SEM. *n* = 3 per group.

As depicted in Figure 6 and Supplementary Figure 9, a single oral dose of cofrogliptin at 1 mg/kg in male beagle dogs led to a substantial decrease in plasma DPP-4 activity. At the 144-h time point, the plasma DPP-4 inhibition rate was 82.3%, with a plasma cofrogliptin concentration of 3.9 ng/mL. When male beagle dogs were administered single oral doses of 3 and 10 mg/kg cofrogliptin, the plasma DPP-4 inhibition rates at 168 h were 82.4% and 94.4%, respectively, with corresponding plasma cofrogliptin concentrations of 6.0 ng/mL and 10.3 ng/mL. Similarly, in female beagle dogs, a single oral dose of 1 mg/kg cofrogliptin significantly reduced plasma DPP-4 activity. At 144 h post-dosing, the inhibition rate was 68.5%, and the plasma cofrogliptin concentration was 3.3 ng/mL. At 168 h after single oral doses of 3 and 10 mg/kg cofrogliptin, the plasma DPP-4 inhibition rates in female beagles were 80.3% and 88.0%, respectively, with corresponding plasma cofrogliptin concentrations of 4.4 ng/mL and 9.0 ng/mL. Collectively, these results highlight the potent inhibitory effect of cofrogliptin on plasma DPP-4 activity in beagle dogs following single-dose administration. Importantly, no significant sex-related differences were observed in the potency or duration of DPP-4 inhibitory effects.

### 3.7 PK-PD relationship of cofrogliptin in inhibiting plasma DPP-4 activity in monkeys

**FIGURE 7:**
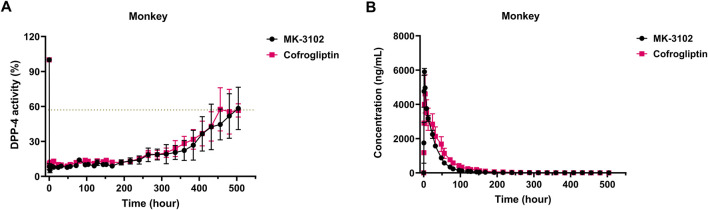
PK-PD relationship of cofrogliptin (10 mg/kg) in inhibiting plasma DPP-4 activity in rhesus monkeys. **(A)** *In vivo* inhibitory activity of cofrogliptin on plasma DPP-4 activity in monkeys. **(B)** Pharmacokinetic profiles of cofrogliptin in monkeys. Data are expressed as mean ± SEM. *n* = 2 per group.

To explore the PK-PD relationship of cofrogliptin in monkeys and compare it with the marketed drug MK3102, we simultaneously evaluated the inhibitory effects of cofrogliptin and MK3102 on rhesus monkey plasma DPP-4 activity and their pharmacokinetic profiles. The results are shown in Figure 7. When orally administered as a single dose of 10 mg/kg, both cofrogliptin and MK3102 significantly inhibited plasma DPP-4 enzyme activity. For cofrogliptin, the inhibition rate of plasma DPP-4 activity reached 90.0% at 0.5 h post-administration, with a corresponding plasma concentration of 1,176.4 ng/mL. This inhibition was sustained at 81.4% even at 288 h (Day 12) after administration, when the plasma concentration was 15.8 ng/mL. At 336 h (Day 14), the inhibition rate remained at 76.2%, with a plasma concentration of 8.7 ng/mL. By 504 h (Day 21), the inhibition rate was 43.4%, and the plasma concentration was 3.0 ng/mL. For MK3102, the inhibition rate of plasma DPP-4 activity also reached 91.6% at 0.5 h post-administration, with a corresponding plasma concentration of 1749.8 ng/mL. At 288 h (Day 12), the inhibition rate was 81.2%, and the plasma concentration was 10.9 ng/mL. At 336 h (Day 14), the inhibition rate was 79.5%, with a plasma concentration of 8.7 ng/mL. By 504 h (Day 21), the inhibition rate was 41.7%, and the plasma concentration was 7.0 ng/mL. These findings demonstrate that both cofrogliptin and MK3102, when administered at a single dose of 10 mg/kg, can effectively inhibit monkey plasma DPP-4 enzyme activity for over 14 days (defined as an inhibition rate of >75%), indicating that they are both long-acting DPP-4 inhibitors.

## 4 Discussion

Inhibition of DPP-4 enhances the levels of active GLP-1 and GIP by preventing their degradation and extending their half-life, thereby restoring glucose homeostasis to normal physiological levels (Pratley and Salsali, 2007). Currently, DPP-4 inhibitors have become a groundbreaking class of anti-hyperglycemic agents and have gained widespread clinical use (American Diabetes Association Professional Practice Committee, 2024; Deacon and Lebovitz, 2016). DPP-4 inhibitors have demonstrated exceptional efficacy and safety profiles in both preclinical studies and clinical practice. However, poor patient adherence to treatment regimens is a significant challenge in the management of type 2 diabetes, often due to the need for frequent dosing and adverse effects. Most currently available oral DPP-4 inhibitors are formulated for once-daily dosing, and two once-weekly options, trelagliptin and omarigliptin, still have notable limitations. For instance, they may be associated with severe adverse reactions or have specific restrictions on use in certain patient populations (Kaku, 2015; Stoimenis et al., 2017). Cofrogliptin is a groundbreaking DPP-4 inhibitor, distinguished by its exceptional potency and prolonged duration of action (Zhang et al., 2020). It stands out as the only DPP-4 inhibitor currently approved for a once-every-two-weeks dosing regimen (Gao et al., 2025). In the present study, we conducted a comprehensive evaluation of the pharmacological profiles of cofrogliptin. We assessed its *in vitro* potency and selectivity, as well as its *in vivo* inhibitory effects on DPP-4 activity in both ICR and *ob/ob* mice. Additionally, we examined the ability of cofrogliptin to reduce glucose excursion during an oral glucose tolerance test in ICR mice and evaluated its impact on blood glucose, serum insulin levels, and DPP-4 activity in *db/db* mice. We also investigated the long-term antidiabetic efficacy of cofrogliptin in *ob/ob* mice. Furthermore, we explored the pharmacokinetics-pharmacodynamics (PK-PD) relationships of cofrogliptin in inhibiting plasma DPP-4 activity in rats, dogs, and monkeys.

Cofrogliptin potently and consistently inhibited DPP-4 across various sources, including recombinant enzymes and serum or plasma from multiple animal species (mice, rats, dogs, and monkeys), with nanomolar IC_50_ values. Its inhibitory activity on DPP-4 was on par with that of trelagliptin and MK3102 (omarigliptin) (Grimshaw et al., 2016). Moreover, cofrogliptin exhibited exceptional *in vitro* target selectivity, as did trelagliptin and MK3102 (PMDA, 2015b; PMDA, 2015a). To further assess its inhibitory effects on DPP-4, cofrogliptin was orally administered to ICR and *ob/ob* mice, and serum DPP-4 activity was subsequently measured at different time points. The results indicated that in ICR mice, at 96 h post-administration, all cofrogliptin-treated groups (1–30 mg/kg) achieved higher inhibition rates of serum DPP-4 activity (24.9%–46.0%) compared to the 30 mg/kg MK3102-treated group (10.7%). In *ob/ob* mice, the medium- and high-dose cofrogliptin groups (10 and 30 mg/kg) also achieved superior inhibition (37.5%–42.3%) compared to the 30 mg/kg MK3102 group (27.6%) at the same time point. The distinctive properties of cofrogliptin imply that it will exert robust and enduring DPP-4 inhibitory effects across preclinical and clinical investigations. Furthermore, compared to MK3102, it appears to possess superior potential for development into a more long-acting therapeutic option for diabetes management.

To validate the aforementioned properties of cofrogliptin, we executed an extensive battery of preclinical assessments to characterize its acute and chronic pharmacological effects on metabolic regulation in both normal and diabetic rodent models. For comparative purposes, we further benchmarked the efficacy of cofrogliptin against that of MK3102. As anticipated, in normal ICR mice, a single oral administration of cofrogliptin (1–10 mg/kg) induced a dose-dependent reduction in blood glucose levels and the area under the blood glucose increment-time curve (AUC_Glu, incre_) following an oral glucose loading of 2.5 g/kg. Notably, the pharmacological effects of cofrogliptin were sustained for 48–72 h post-administration, whereas the effects of 10 mg/kg MK3102 were confined to a maximum duration of 48 h. In ICR mice, the minimum effective dose of cofrogliptin for reducing glucose excursion was 1 mg/kg. At this dose, a maximal reduction of 29.75% in AUC_Glu, incre_ and a 44.59% increase in active GLP-1 levels were observed. When the dose was increased to 3 mg/kg, cofrogliptin induced a maximal reduction of 35.89% in AUC_Glu, incre_ and a 110.29% increase in active GLP-1 levels. This efficacy was comparable to that of 10 mg/kg MK3102, which achieved a maximal reduction of 37.65% in AUC_Glu, incre_ and a 102.11% increase in active GLP-1 levels (Supplementary Tables 5, 6). These findings suggest that cofrogliptin demonstrated a more potent glucose-lowering efficacy than MK3102, and this superior pharmacological activity aligns with its favorable PK profiles observed in mice. Specifically, the enhanced PK performance of cofrogliptin stems from a key factor: the structural modification of MK3102, namely, the introduction of a trifluoromethyl group at the 6-position of its tetrahydropyran ring, which results in a notably lower clearance rate in mice (2.57 mL·min^-1^·kg^-1^, compared to 7.39 mL·min^-1^·kg^-1^ for MK3102) (Zhang et al., 2020). The acute glucose-lowering effect of cofrogliptin was further assessed in *db/db* mice, a genetic model of type 2 diabetes characterized by hyperglycemia, hyperinsulinemia, hyperglucagonemia, and severe systemic insulin resistance (Kleinert et al., 2018; Suriano et al., 2021). During the oral glucose tolerance test (OGTT) in *db/db* mice, a single oral dose of 10 mg/kg cofrogliptin significantly reduced blood glucose levels, with its therapeutic effects lasting for over 58 h. Additionally, this dose of cofrogliptin increased serum insulin levels and robustly inhibited serum DPP-4 activity. Collectively, these results demonstrate that cofrogliptin improved glucose tolerance in both normal and diabetic animal models by elevating active GLP-1 and serum insulin levels via the inhibition of DPP-4 activity. These findings are consistent with previous studies using other DPP-4 inhibitors in glucose-intolerant animals, such as high-fat-diet/streptozotocin (HFD/STZ)-induced diabetic mice (Kim et al., 2016), high-fat-diet-induced obese mice (Fan et al., 2022), and Zucker *fa/fa* rats (Tanaka-Amino et al., 2008; Jain et al., 2017).

Multiple studies have shown that long-term DPP-4 inhibition effectively ameliorates metabolic disorders and preserves pancreatic functions in diabetic animal models (Fan et al., 2022; Thomas et al., 2009; Furuta et al., 2010). In this study, we examined the antidiabetic effects of long-term DPP-4 inhibition by cofrogliptin in *ob/ob* mice. Given that a single oral dose of cofrogliptin (1–30 mg/kg) in *ob/ob* mice resulted in a DPP-4 inhibition rate of 42.5%–51.2% at 72 h post-dosing (Figure 3B), an administration frequency of once every 3 days was chosen for the chronic study to ensure sustained adequate inhibition of DPP-4 activity throughout the experiment. As reported for other DPP-4 inhibitors (Thomas et al., 2009; Fukuda-Tsuru et al., 2012), cofrogliptin significantly reduced random and fasting blood glucose levels. The glucose-lowering efficacy of cofrogliptin at 10 mg/kg was comparable to that of MK3102 at 30 mg/kg. Chronic administration of cofrogliptin also reversed the hyperlipidemic phenotype in *ob/ob* mice, as evidenced by reduced serum triglycerides (TG), free fatty acids (FFA), and hepatic TG accumulation. These findings align with previously reported effects of DPP-4 inhibitors on lipid metabolism. For instance, teneligliptin has been shown to reduce serum TG and visceral fat mass in high-fat-diet-induced obese mice, decrease plasma TG and FFA levels in Zucker fatty rats, and attenuate hepatic TG and FFA accumulation in *ob/ob* mice (Fukuda-Tsuru et al., 2012; Wang et al., 2020; Nakamura et al., 2017). Notably, cofrogliptin at a mere 1 mg/kg outperformed MK3102 at 30 mg/kg in ameliorating lipid metabolism disorders (Supplementary Tables 8, 9). Moreover, similar to vildagliptin and alogliptin (Moritoh et al., 2008; Fan et al., 2022), cofrogliptin enhanced pancreatic beta-cell function in *ob/ob* mice. The above results suggest that cofrogliptin may exert superior effects in type 2 diabetic patients with concurrent lipid dysregulation.

Establishing a robust PK-PD relationship is essential for translating non-clinical findings into clinical development. To this end, we characterized the plasma concentration-response relationship of DPP-4 inhibition by cofrogliptin in rats, dogs, and monkeys. In the monkey study, we further benchmarked cofrogliptin against MK3102 to contrast their respective PK-PD profiles. In rats, single oral doses of cofrogliptin (3–30 mg/kg) produced a plasma half-life of 19–57 h (Supplementary Table 12), nearly 2- to 5-fold longer than the 11 h measured for MK3102 (Chen et al., 2015; Biftu et al., 2014). At an equivalent oral dose, cofrogliptin also achieved greater systemic exposure, as reflected by an AUC value that exceeded that of MK3102 (Supplementary Table 12). Additionally, we observed significant sex differences in both the plasma concentrations of cofrogliptin and its DPP-4 inhibitory activity. Male rats maintained 90% DPP-4 inhibition for approximately 72 h, whereas female rats sustained this level of DPP-4 inhibition for at least 168 h. Correspondingly, at the 168-h time point, the plasma concentration of cofrogliptin in female rats exceeded that in male rats by more than 118-fold. In a previous rat PK study, we observed that following intravenous administration of cofrogliptin at a dose of 3 mg/kg, the clearance rates in male and female rats were 0.472 and 0.125 mL·min^-1^·kg^-1^, respectively. Additionally, after oral administration of cofrogliptin at 3 mg/kg, the maximum plasma concentrations (C_max_) in male and female rats were 9,460 and 10,698 ng·mL^-1^, respectively. These findings collectively suggest that sex differences in metabolism may represent the primary driver underlying the significant sex-related disparities in the *in vivo* PK profiles of cofrogliptin. However, the specific underlying mechanisms remain to be elucidated. In dogs, after a single oral administration, cofrogliptin and MK3102 demonstrated comparable plasma half-lives, and no sex-related disparities were discernible (Supplementary Table 13) (Chen et al., 2015). A single dose of cofrogliptin maintained 90% DPP-4 inhibition for over 144 h. In monkeys, after a single oral dose of 10 mg/kg, cofrogliptin and MK3102 showed comparable half-lives and AUCs (Supplementary Table 14). Their DPP-4 inhibitory activity was also similar across all post-dose time points, with approximately 80% inhibition maintained through 336 h.

Based on the prediction using allometric scaling from the PK profiles across four species (mice, rats, dogs, and monkeys), cofrogliptin is projected to have superior human PK characteristics compared to MK3102. As previously reported, at clinically effective doses, cofrogliptin (10 mg) achieved a C_max_ comparable to that of MK3102 (25 mg). However, its plasma half-life was 1.66–2.84-fold longer (Shi et al., 2024; Bai et al., 2023; Addy et al., 2016; Krishna et al., 2016). This discrepancy aligns with their respective dosing schedules: cofrogliptin is administered once every 2 weeks, whereas MK3102 is dosed weekly. Notably, we have developed a population pharmacokinetics and pharmacodynamics (PopPKPD) model to simulate the plasma concentration–time and DPP-4 inhibition rate–time profiles of cofrogliptin across a multiple-dose range of 5–150 mg in humans. Results revealed that bi-weekly administration of 10 mg cofrogliptin yielded a DPP-4 inhibition rate exceeding 80% over the interdosing interval, with the PK and PD profiles simulated based on the covariate characteristics of the multiple ascending dose (MAD) study population. Importantly, comparative analysis of model-simulated data *versus* clinical data derived from the MAD trial demonstrated robust external predictive performance, as predicted values were in close agreement with the observed data throughout the study period (Cui et al., 2023). Therefore, the aforementioned study findings offer compelling evidence to support the bi-weekly dosing frequency of cofrogliptin in patients with type 2 diabetes.

## 5 Conclusion

These findings establish cofrogliptin as a next-generation DPP-4 inhibitor that combines exceptional potency and selectivity with an extended pharmacokinetic and pharmacodynamic profile, supporting once-every-two-week dosing. As a result, cofrogliptin stands out as a highly effective and long-acting therapeutic option for the sustained management of type 2 diabetes, and these key traits in turn enhance patient adherence while expanding its combination therapy potential.

## Data Availability

The original contributions presented in the study are included in the article/Supplementary Material, further inquiries can be directed to the corresponding author.

## Author disclaimer

All authors are employees of Haisco Pharmaceutical Group Co., Ltd. Haisco has developed cofrogliptin as a treatment for diabetes and is selling the drug under the trade name beichangping®. However, we hereby confirm that the studies were conducted under the independent ethical oversight of the Institutional Animal Care and Use Committee (IACUC) of Shanghai Institute of Materia Medica, Chinese Academy of Sciences (Shanghai, China), Soochow University (Suzhou, China), 3D BioOptima (Suzhou) Co., Ltd (China), or Sichuan Plamei Biotechnology Co., Ltd. (Chengdu, China). Furthermore, all experimental processes and data collection were subjected to rigorous quality control protocols to ensure the integrity and objectivity of the results.
