# Supplementary material for: Preclinical pharmacological profiles of cofrogliptin, a novel and bi-weekly DPP-4 inhibitor
Source: Front Pharmacol. 2026 Jan 5;16:1702101. doi: 10.3389/fphar.2025.1702101 (PMC12812960; doi:10.3389/fphar.2025.1702101)
Supplement: Supplementary file 1 [file Supplementaryfile1.docx]

Supplemental material

**Supplemental materials and methods**

**Animals**

C57BL/6J and Institute of Cancer Research (ICR) mice were provided by Shanghai Lingchang Biotechnology Co., Ltd. (Shanghai, China). B6.V-Lepob/J (*ob/ob*, stock number 000632) mice, wild-type B6 mice and B6.Cg-m +/+ Leprdb/J (*db*/*db*, stock number 000697) mice were obtained from The Jackson Laboratory (Maine, USA) and subsequently maintained and bred at Shanghai Institute of Materia Medica, Chinese Academy of Sciences (Shanghai, China). Sprague-Dawley (SD) rats were sourced from Shanghai Xipur Bikai Laboratory Animal Co., Ltd. (China) or Beijing Vital River Laboratory Animal Technology Co., Ltd. (China) and were subsequently housed and bred at Soochow University (China). Beagle dogs were acquired from Beijing Marshall Biotechnology Co., Ltd. (China) and were bred at 3D BioOptima (Suzhou) Co., Ltd. (China). Rhesus monkeys were provided by Sichuan Plamei Biotechnology Co., Ltd. (Chengdu, China), where they were also bred.

**Reagents and drugs**

Verapamil was obtained from National Institute for the Control of Pharmaceutical and Biological Products (Beijing, China). H-Ala-Pro-AFC was purchased from Bachem (Bubendorf, Switzerland). GLP-1(7-36) active ELISA kit was from Millipore Corporation (MA, USA). Normal goat serum was provided by Beijing Dingguo Biotechnology Co., Ltd. (China). Guinea pig polyclonal to insulin, Rb pAb to glucagon, and goat polyclonal antibody to guinea pig IgG H&L (Texas Red) were purchased from Abcam plc (Cambridge, UK). Dylight 488-conjugated goat anti-rabbit IgG H&L was obtained from MultiSciences (Lianke) Biotech Co., Ltd. (Hangzhou, China). Triglyceride (TG) assay kit was from Zhejiang Dongou Diagnostic Products Co., Ltd. (Wenzhou, China).

**Enzyme inhibition assay buffers**

For the recombinant DPP-4 enzyme inhibition assay, the buffer was 50 mM Tris (pH 7.5). For the endogenous DPP-4 inhibition assay, as well as the recombinant DPP-8 and DPP-9 enzyme inhibition assays, the buffer comprised 25 mM HEPES, 140 mM NaCl, 1% BSA, and 80 mM MgCl_2_ (pH 7.8). This buffer was also used to evaluate the effects of cofrogliptin and MK3102 on DPP-4 activity in ICR and *ob/ob* mice.

***In vitro* SafetyScreen panel**

*In vitro* off-target pharmacological activities of cofrogliptin were evaluated on 127 targets using a SafetyScreen panel and the corresponding methods can be found at <https://apac.eurofinsdiscovery.com/>.

**Effects on plasma active GLP-1 levels in ICR mice**

A total of 170 male ICR mice (25–29 g) were utilized in this assay. After an acclimation period, 160 mice were randomly allocated into four major groups according to body weight, corresponding to four time points (24, 48, 72, and 96 hours post-administration). Each major group was further divided into four subgroups, which included the 1 mg/kg cofrogliptin group, the 3 mg/kg cofrogliptin group, the 10 mg/kg cofrogliptin group, and the 10 mg/kg MK3102 group. Mice in each subgroup were orally administered the respective doses of cofrogliptin or MK3102 24, 48, 72, or 96 hours in advance, depending on their assigned time point. Additionally, the remaining 10 mice were assigned to the vehicle group and orally administered 1% HPMC (vehicle) 48 hours in advance. All animals were fasted overnight and then orally administered 2.5 g/kg glucose. Blood samples were collected into centrifuge tubes at 15 minutes after glucose administration. The plasma was separated from the blood samples and used to measure the levels of active GLP-1 [7-36 amide]. The levels of active GLP-1 in plasma were determined using an enzyme-linked immunoassay (ELISA) kit.

**Serum glucose level measurement**

The glucose measurement was meticulously performed in strict accordance with the manufacturer's instructions of the glucose assay kit (Shanghai Rongsheng Biotech Co., Ltd.). Briefly, equal volumes of reagents R1 and R2 were thoroughly mixed to prepare the enzyme working solution, which was promptly stored at 4 °C for subsequent use. Then, 2.5 μL of each serum sample was pipetted into separate test tubes. In addition, a blank tube containing 2.5 μL of double-distilled water and a standard tube with 2.5 μL of glucose standard solution were prepared. Exactly 500 μL of the pre-prepared enzyme working solution was then added to each tube. After thorough mixing, the tubes were incubated in a 37 °C water bath for 15 minutes. Absorbance readings were taken at 505 nm, with the blank tube serving as the reference for zero adjustment. Finally, the serum glucose concentration was calculated using the provided formula: OD_serum_/OD_standard_ × 5.55. Here, OD_serum_ denotes the absorbance reading of the serum sample, while OD_standard_ represents the absorbance reading of the glucose standard solution.

**Quantification of insulin concentration in serum**

The procedure was conducted following the manufacturer's instructions for the ultra-sensitive mouse insulin ELISA kit (CrystalChem).

**Determination of hepatic triglyceride content**

Following mouse dissection, the liver was promptly excised and flash-frozen in liquid nitrogen to preserve tissue integrity. For triglyceride extraction, a precisely measured liver sample was homogenized in 2 mL of extraction buffer (n-heptane/isopropanol/Tween 80 = 3/2/0.01). The homogenate was centrifuged at 1500 × g for 15 min at 4 °C to separate its components. The supernatant was carefully collected and transferred to a clean glass tube, then evaporated to dryness under controlled conditions. Next, 1 mL of triglyceride assay reagent was added to each dried sample, followed by incubation at 37 °C for 15 min. For spectrophotometric analysis, 150 μL of the reaction mixture was aliquoted into a 96-well microplate. The absorbance of the samples was measured at two specific wavelengths, 546 nm and 660 nm using an automated fluorescent plate reader (FlexStation III, Molecular Device, CA, USA). Hepatic triglyceride content was then calculated using an appropriate standard curve.

**Immunofluorescence staining-based analysis of insulin and glucagon expression in pancreatic tissue**

During the dissection of mice, the pancreas was meticulously excised and immediately immersed in a 10% formaldehyde fixative solution to ensure optimal preservation of the tissue architecture (Mu et al., 2006). Subsequently, the pancreatic tissues were processed for embedding, and 3-μm-thick sections were precisely prepared for immunofluorescence staining targeting insulin and glucagon. The pancreatic sections were subjected to routine deparaffinization and rehydration through a series of sequential incubations: three 10-minute immersions in xylene, followed by three 10-minute incubations in absolute ethanol, a 5-minute incubation in 95% ethanol, a 5-minute incubation in 85% ethanol, and a final 5-minute incubation in 75% ethanol. Following this, the slides were thoroughly washed twice with TBS-T buffer (5 minutes each), treated with 3% hydrogen peroxide for 10 minutes to effectively quench endogenous peroxidase activity, and then rinsed once with TBS-T (5 minutes). To enhance epitope exposure, antigen retrieval was performed by immersing the slides in an antigen retrieval solution, microwaving until boiling, allowing the slides to cool to room temperature, and repeating the boiling-cooling cycle once. The slides were then washed twice with TBS-T (5 minutes each) and incubated with a 5% normal goat serum blocking solution for 45 minutes to minimize nonspecific binding. After the blocking solution was carefully removed, the sections were incubated overnight at 4 °C with primary antibody working solutions, specifically insulin at a 1:50 and glucagon at a 1:100, to facilitate specific antigen-antibody interactions. The following day, the slides were washed three times with TBS-T (5 minutes each) to remove unbound primary antibodies and then incubated with a 10% normal goat serum blocking solution for 30 minutes to further reduce nonspecific binding. Following the removal of the blocking solution, secondary antibody working solutions were applied—insulin at a 1:200 and glucagon at a 1:100—and the slides were incubated at 37 °C for 60 minutes to ensure robust binding of secondary antibodies to the primary antibodies. The slides were then washed three times with TBS-T (5 minutes each) to remove unbound secondary antibodies and mounted with an anti-fade mounting medium to preserve the immunofluorescence signals. Finally, the immunofluorescence signals were visualized and imaged using a laser scanning confocal microscope (Olympus FV1000-SIM, Japan), capturing high-resolution images of insulin and glucagon expression within the pancreatic tissue.

**Qualification of cofrogliptin in rat and dog plasma**

After thawing the plasma samples on ice, 20 µL (for rats) or 30 µL (for dogs) of plasma was transferred into a centrifuge tube. Subsequently, 400 µL (for rats) or 300 µL (for dogs) of an internal standard solution containing verapamil (5 ng/mL) was added to the plasma. The mixture was vortexed for 0.2 minutes and then centrifuged at 13,000 rpm for 8 minutes at 4 °C. A volume of 50 µL (for rats) or 70 µL (for dogs) of the supernatant was collected and mixed with 100 µL (for rats) or 70 µL (for dogs) of water. This mixture was then placed into an injection bottle for subsequent analysis. The analysis was performed using a high-performance liquid chromatography-tandem mass spectrometry (HPLC-MS/MS) system, which consisted of a LC-20AD HPLC (Shimadzu, Japan) coupled to an API4000 triple quadrupole tandem mass spectrometer (Applied Biosystems, USA) via an electrospray ionization (ESI) source. Chromatographic separation was achieved using a reverse-phase column (Agilent Zorbax XDB-C18, 50 × 2.1 mm, 5 µm) with a gradient elution process as described in the table below. The mobile phase A was composed of 0.1% formic acid in water, while mobile phase B was composed of 0.1% formic acid in acetonitrile. The injection volume was set to 5 µL. Quantification was conducted using multiple reaction monitoring (MRM) mode. The mass spectrometer was operated with the following parameters: spray voltage at 5.5 kV, ion source temperature at 500 °C, nebulizing gas (GS1) pressure at 50 psi, auxiliary gas (GS2) pressure at 50 psi, curtain gas pressure at 20 psi, and collision gas pressure at 12 psi. The collision-induced dissociation voltages were set at 37 eV for cofrogliptin and 42 eV for verapamil. The MRM transitions used for quantification were m/z 467.2→173.2 for cofrogliptin and m/z 455.2→165.1 for verapamil.

| **Time (min)** | **Flow rate (mL/min)** | **Mobile phase B (%)** |
| --- | --- | --- |
| 0.50 | 0.40 | 20 |
| 1.20 | 0.40 | 95 |
| 2.20 | 0.40 | 95 |
| 2.21 | 0.40 | 20 |
| 3.50 | 0.40 | Stop |

**Qualification of MK-3102 and cofrogliptin in monkey plasma**

After thawing the plasma samples on ice, 30 µL of the plasma was transferred into a centrifuge tube. Next, 200 µL of an internal standard solution with verapamil at a concentration of 300 ng/mL was added to the plasma. The mixture was vortexed for 0.2 minutes to ensure thorough mixing and then centrifuged at 13,000 rpm for 10 minutes at 4 °C. Subsequently, 180 µL of the supernatant was carefully collected and transferred into an injection bottle for further analysis. The analysis was carried out using a high-performance liquid chromatography-tandem mass spectrometry (HPLC-MS/MS) system. This system comprised a LC-20AD HPLC (Shimadzu, Japan) coupled to an API4000 triple quadrupole tandem mass spectrometer (Applied Biosystems, USA) via an electro-spray ionization (ESI) source. Chromatographic separation was achieved using a reverse-phase column (Agilent Zorbax XDB-C18, 50 × 2.1 mm, 5 µm) with a gradient elution process, details of which are presented in the following table. The mobile phase A was composed of 0.1% formic acid in water, while mobile phase B was composed of 0.1% formic acid in methanol. Quantification was performed using multiple reaction monitoring (MRM) mode. The mass spectrometer was operated with the following parameters: spray voltage at 5.5 kV, ion source temperature at 500 °C, nebulizing gas (GS1) pressure at 50 psi, auxiliary gas (GS2) pressure at 50 psi, curtain gas pressure at 20 psi, and collision gas pressure at 12 psi. The collision-induced dissociation voltages were set at 40 eV for MK3102, 36 eV for cofrogliptin, and 37 eV for verapamil. The MRM transitions used for quantification were m/z 399.2→153.3 for MK3102, m/z 467.2→173.2 for cofrogliptin, and m/z 455.2→165.1 for verapamil.

| **Compound** | **Time (min)** | **Flow rate (mL/min)** | **Mobile phase B (%)** |
| --- | --- | --- | --- |
| MK3102 | 0.5 | 0.3 | 15 |
|  | 0.8 | 0.3 | 95 |
|  | 1.2 | 0.3 | 95 |
|  | 1.3 | 0.6 | 95 |
|  | 2.5 | 0.6 | 95 |
| Cofrogliptin | 0.9 | 0.4 | 5 |
|  | 1 | 0.4 | 95 |
|  | 1.2 | 0.4 | 95 |
|  | 1.3 | 0.6 | 95 |
|  | 2 | 0.6 | 95 |

**Detection of DPP-4 enzymatic activity in monkey plasma**

A plasma sample with a volume of 40 μL was combined with 10 μL of H-Ala-Pro-AFC substrate at a concentration of 0.2 mM. The mixture was then incubated at room temperature for 5 minutes. Following incubation, the fluorescence intensity (FI) was measured using an automated fluorescent plate reader (EnVision, Perkin Elmer, USA) with excitation at 405 nm and emission at 535 nm. The DPP-4 activity was determined using the following formula: DPP-4 activity (%) = FI_t_ / FI_0h_ × 100%, where FI_t_ indicates the fluorescence intensity at different time points after drug administration, and FI_0h_ represents the fluorescence intensity of samples before drug administration.

**
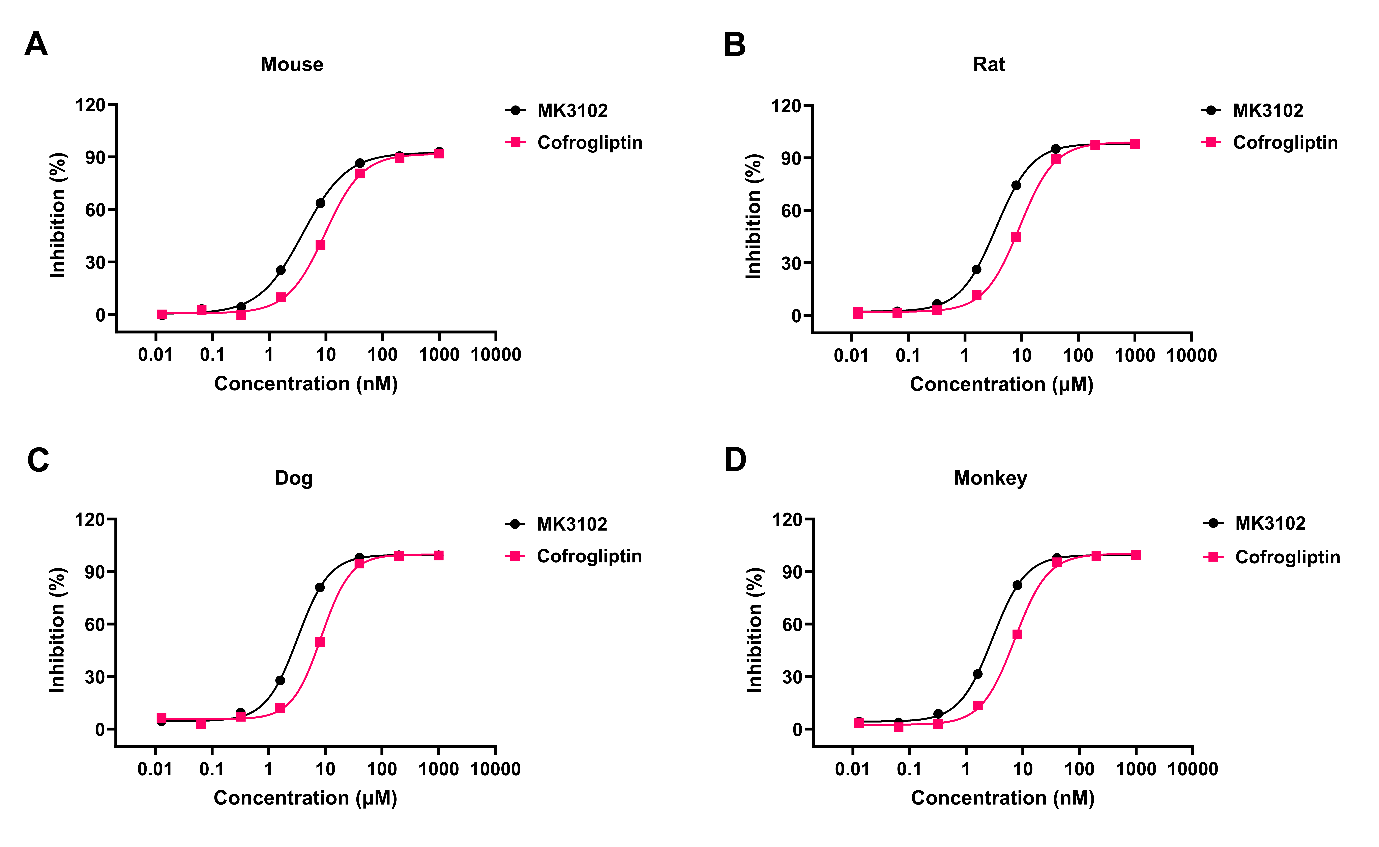
**

**Supplemental Figure 1.** *In vitro* potency of cofrogliptin in endogenous DPP-4 inhibition assays. Inhibitory activity of cofrogliptin on endogenous DPP-4 in mouse plasma (A), rat plasma (B), dog serum (C), and monkey serum (D). Data are expressed as mean ± SEM. n=3 per group.

**
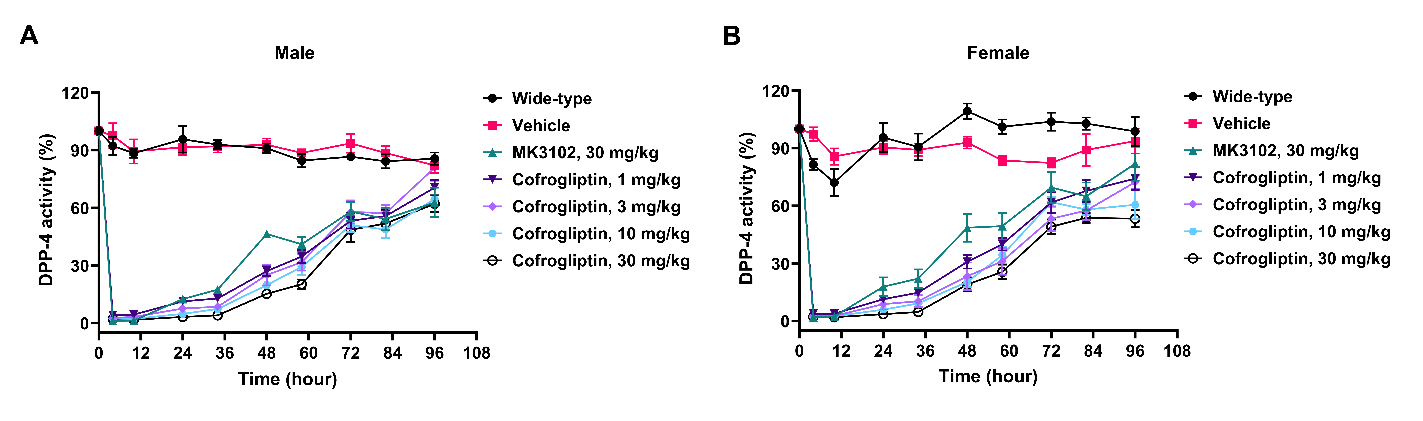
**

**Supplemental Figure 2.** *In vivo* inhibitory activity of cofrogliptin on serum DPP-4 activity in male (A) and female (B) *ob/ob* mice. Data are expressed as mean ± SEM. n=5 per group.

**
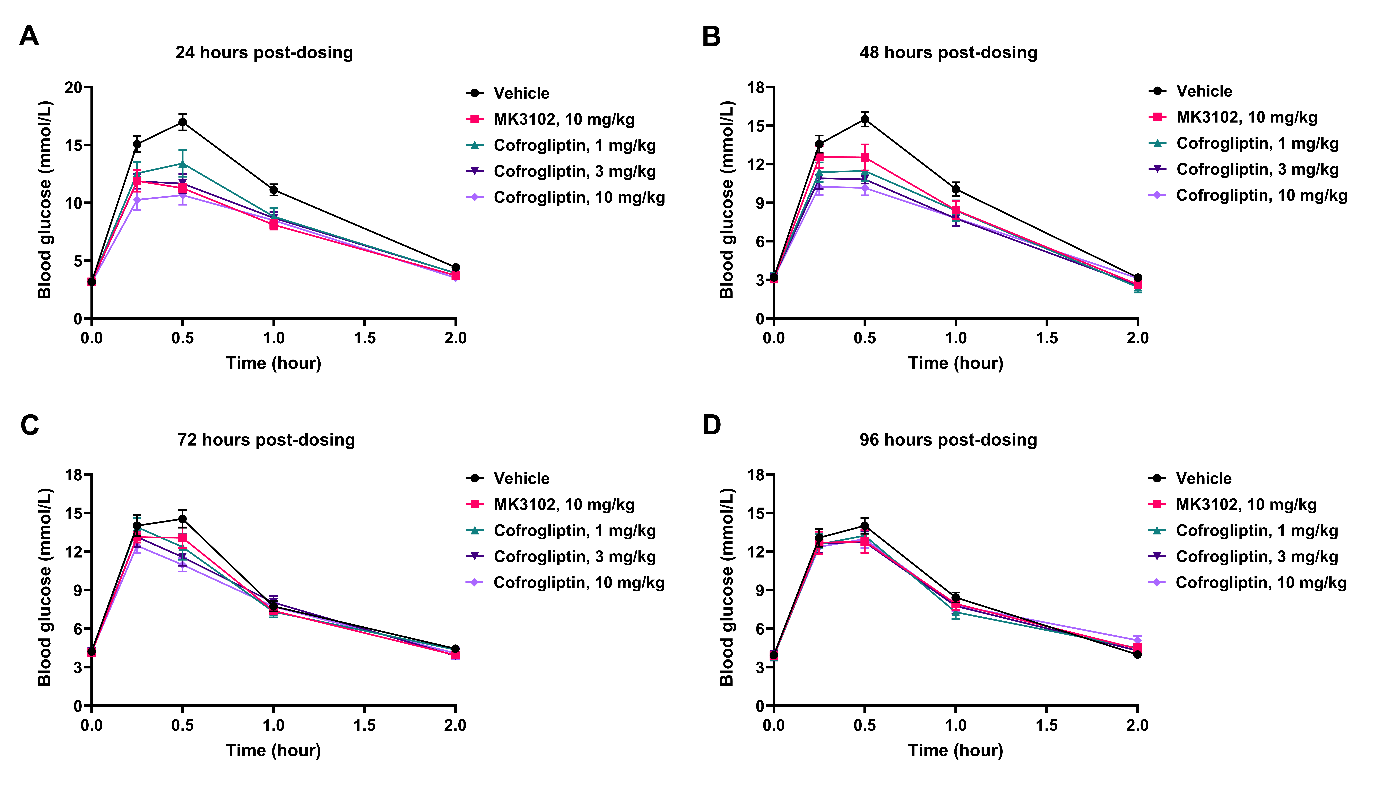
**

**Supplemental Figure 3.** Effects of cofrogliptin on blood glucose levels during oral glucose tolerance test in ICR mice at 24, 48, 72 and 92 hours post-dosing. Data are expressed as mean ± SEM. n=10 per group.

**
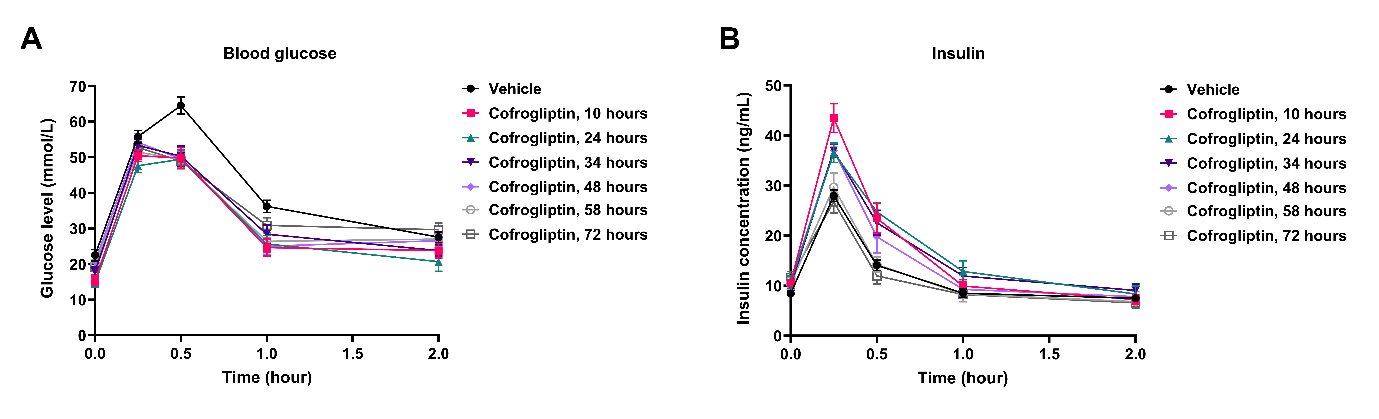
**

**Supplemental Figure 4.** Effects of cofrogliptin on blood glucose (A), and serum insulin levels (B) in *db/db* mice. Data are expressed as mean ± SEM. n=10 per group.

**
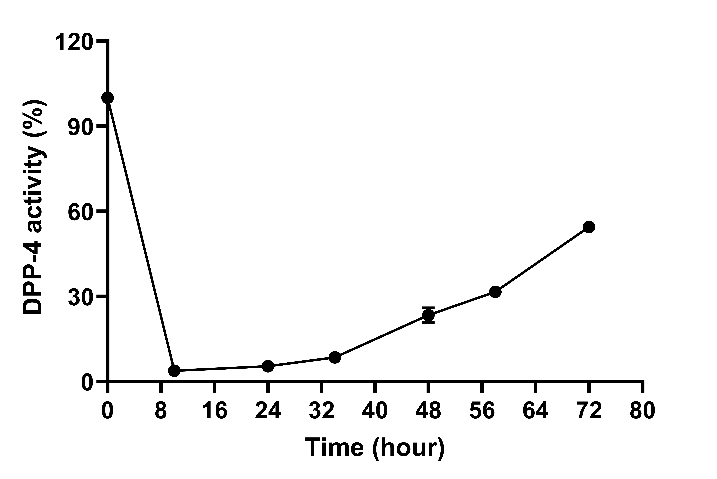
**

**Supplemental Figure 5.** Effects of cofrogliptin (10 mg/kg) on serum DPP-4 activity in *db/db* mice. Data are expressed as mean ± SEM. n=10 per group.

**
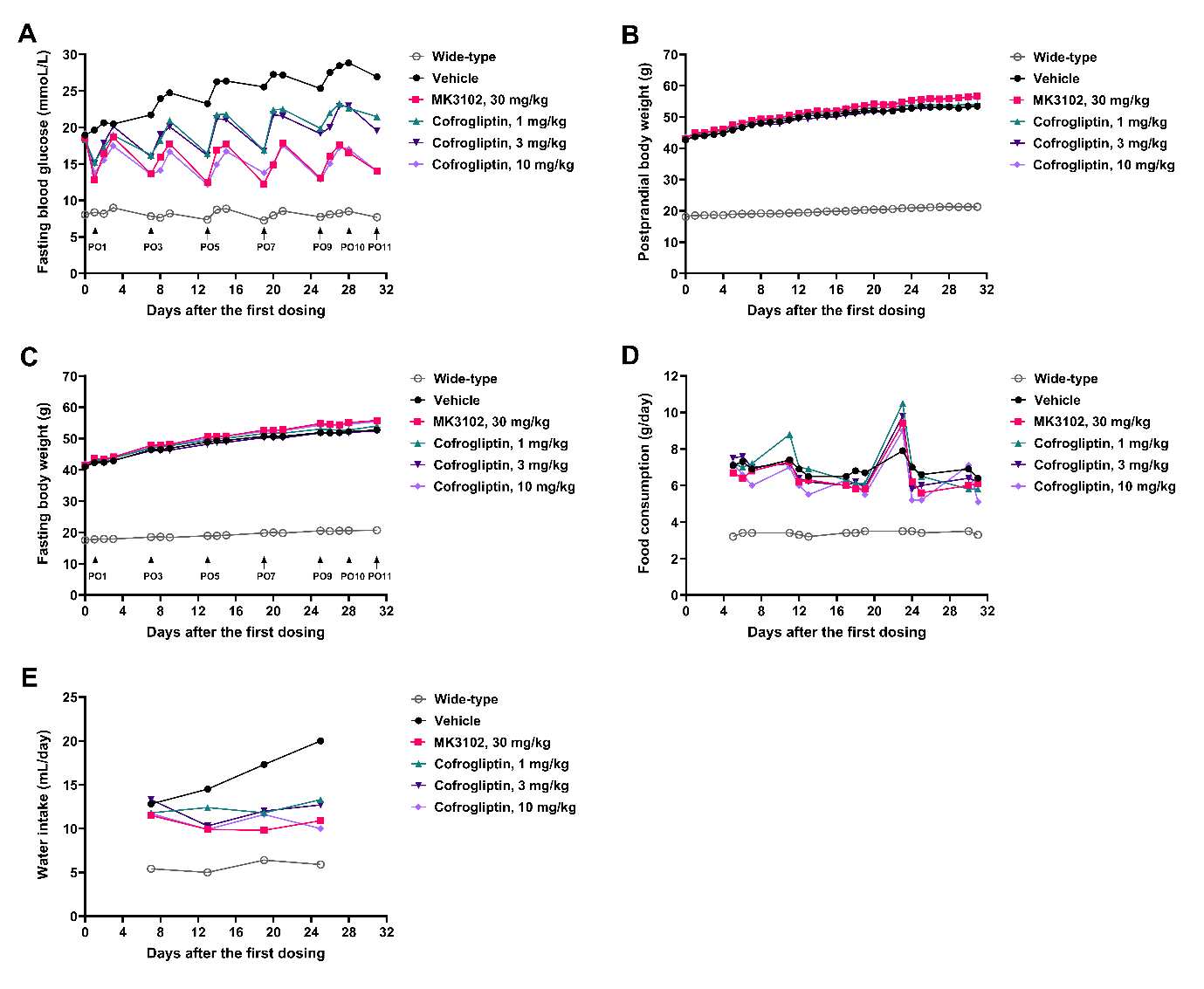
**

**Supplemental Figure 6.** Effects of long-term oral administration of cofrogliptin on fasting blood glucose (A), postprandial body weight (B), fasting body weight (C), food consumption (D), and water intake (E). Data are expressed as mean ± SEM. n=12 per group.


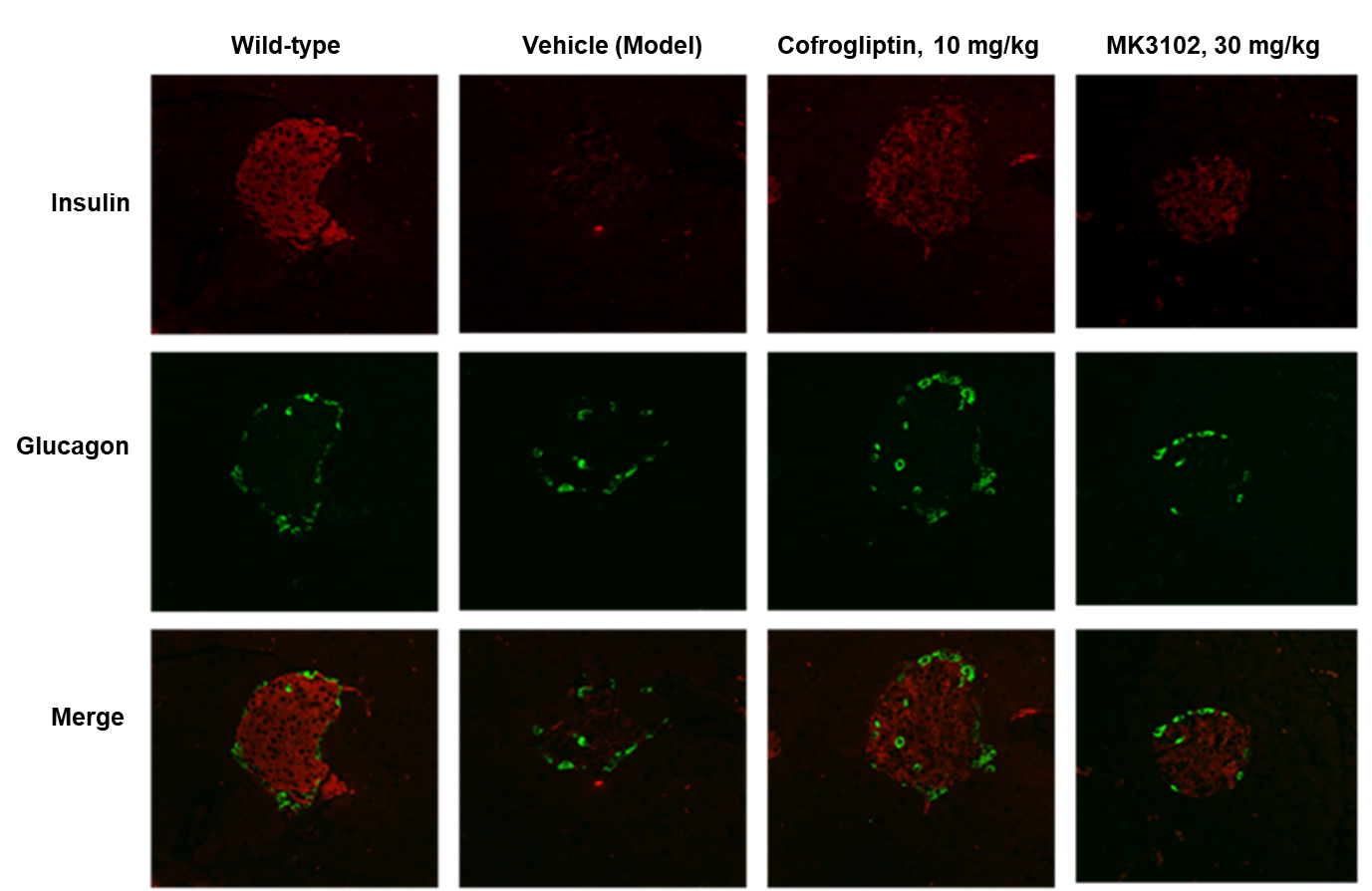


**Supplemental Figure 7.** Effects of chronic cofrogliptin administration on insulin and glucagon expression in pancreatic tissue. Pancreatic sections were stained with anti-insulin (red) or anti-glucagon (green) antibodies. Original magnification 🞨40.

**
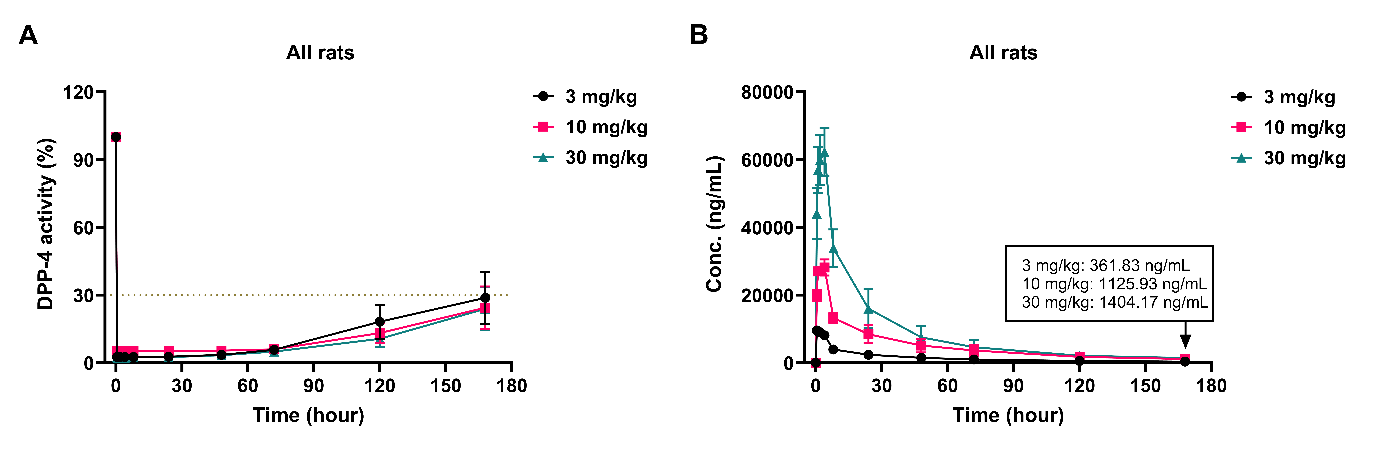
**

**Supplemental Figure 8.** PK-PD relationship of cofrogliptin in inhibiting plasma DPP-4 activity in SD rats. (A) *In vivo* inhibitory activity of cofrogliptin on plasma DPP-4 activity in rats. (B) Pharmacokinetic profiles of cofrogliptin in rats. Data are expressed as mean ± SEM. n=6 per group.

**
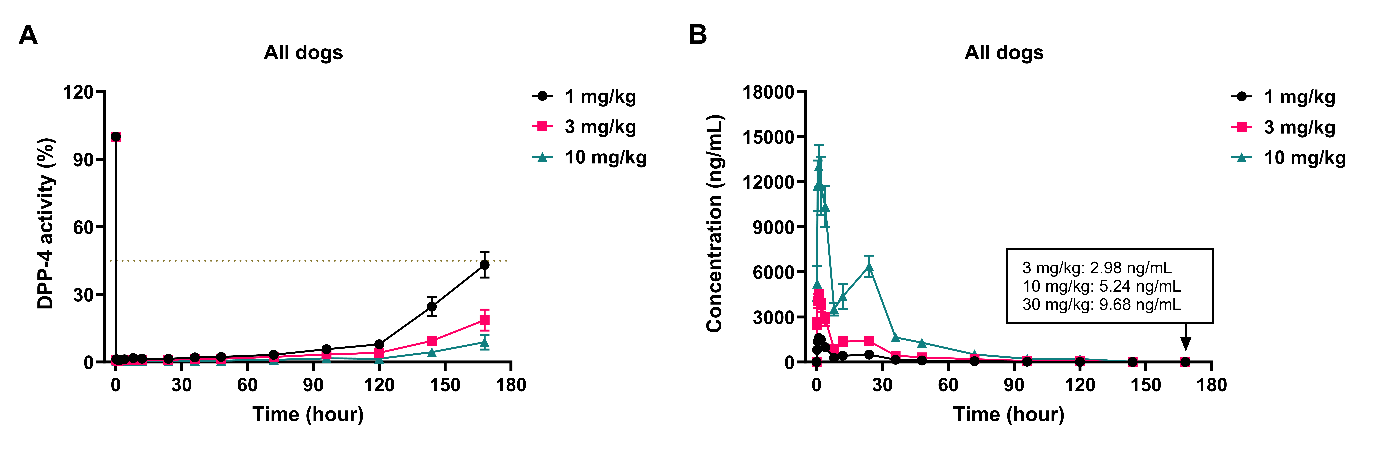
**

**Supplemental Figure 9.** PK-PD relationship of cofrogliptin in inhibiting plasma DPP-4 activity in Beagle dogs. (A) *In vivo* inhibitory activity of cofrogliptin on plasma DPP-4 activity in dogs. (B) Pharmacokinetic profiles of cofrogliptin in dogs. Data are expressed as mean ± SEM. n=6 per group.

**Supplemental Table 1.** Metabolic profiles of *db*/*db* mice prior to drug administration in oral glucose tolerance test (OGTT)

| **Group** | | **Body weight (g)** | **Fasting blood glucose (mmol/L)** | **Serum insulin**  **(ng/mL)** |
| --- | --- | --- | --- | --- |
| Vehicle | -- | 41.40 ± 1.20 | 20.42 ± 1.34 | 10.66 ± 1.00 |
| Cofrogliptin,  10 mg/kg | 10 h | 41.20 ± 0.50 | 20.23 ± 1.26 | 10.58 ± 1.28 |
| Cofrogliptin,  10 mg/kg | 24 h | 41.30 ± 0.70 | 20.37 ± 1.32 | 10.53 ± 1.21 |
| Cofrogliptin,  10 mg/kg | 34 h | 41.10 ± 0.80 | 20.30 ± 1.23 | 10.43 ± 1.19 |
| Cofrogliptin,  10 mg/kg | 48 h | 40.90 ± 1.00 | 20.20 ± 1.34 | 10.47 ± 1.10 |
| Cofrogliptin,  10 mg/kg | 58 h | 40.90 ± 0.90 | 20.45 ± 1.24 | 10.50 ± 0.84 |
| Cofrogliptin,  10 mg/kg | 72 h | 41.40 ± 1.00 | 20.47 ± 1.23 | 10.48 ± 0.76 |

Note: Data are expressed as mean ± SEM. n=10 per group.

**Supplemental Table 2.** The detailed grouping information and baseline parameters of the animals in the long-term antidiabetic efficacy evaluation of cofrogliptin in *ob/ob* mice

| **Group** | **Postprandial**  **body weight (g)** | **Fasting**  **body weight (g)** | **Random**  **blood glucose (mmoL/L)** | **Fasting**  **blood glucose (mmoL/L)** | **HbA1c**  **(%)** | **Serum insulin (ng/mL)** |
| --- | --- | --- | --- | --- | --- | --- |
| Wide-type | 18.1 ± 0.5 | 17.5 ± 0.5 | 8.21 ± 0.35 | 8.05 ± 0.36 | 3.94 ± 0.04 | 0.41 ± 0.05 |
| Vehicle | 42.7 ± 1.2 | 41.0 ± 1.1 | 18.61 ± 0.68 | 18.96 ± 0.67 | 5.04 ± 0.20 | 9.24 ± 0.63 |
| MK3102,  30 mg/kg | 43.1 ± 1.0 | 41.5 ± 1.0 | 18.91 ± 0.85 | 18.57 ± 0.70 | 5.03 ± 0.20 | 9.35 ± 1.04 |
| Cofrogliptin,  1 mg/kg | 43.2 ± 1.2 | 41.5 ± 1.1 | 18.90 ± 0.82 | 18.34 ± 0.58 | 5.00 ± 0.15 | 9.25 ± 0.55 |
| Cofrogliptin,  3 mg/kg | 42.5 ± 1.0 | 41.0 ± 1.0 | 18.80 ± 0.92 | 18.26 ± 0.74 | 5.06 ± 0.16 | 9.16 ± 0.62 |
| Cofrogliptin,  10 mg/kg | 43.6 ± 1.2 | 41.9 ± 1.2 | 18.67 ± 0.73 | 19.14 ± 0.70 | 4.99 ± 0.15 | 9.56 ± 0.72 |

Note: Data are expressed as mean ± SEM. n=12 per group.

**Supplemental Table 3.** The experimental schedule for the long-term antidiabetic efficacy evaluation of cofrogliptin in *ob/ob* mice

| **Days** | **Dosing** | **Random blood glucose** | **Fasting blood glucose** | **Postprandial body weight** | **Fasting body weight** | **Food consumption** | **Water intake** | **Dissection** |
| --- | --- | --- | --- | --- | --- | --- | --- | --- |
| 1 | ● | ★ | ☆ | ▲ | △ |  |  |  |
| 2 |  | ★ | ☆ | ▲ | △ |  |  |  |
| 3 |  | ★ | ☆ | ▲ | △ |  |  |  |
| 4 | ● | ★ |  | ▲ |  |  |  |  |
| 5 |  |  |  | ▲ |  | ○ |  |  |
| 6 |  |  |  | ▲ |  | ○ |  |  |
| 7 | ● | ★ | ☆ | ▲ | △ | ○ | ● |  |
| 8 |  | ★ | ☆ | ▲ | △ |  |  |  |
| 9 |  | ★ | ☆ | ▲ | △ |  |  |  |
| 10 | ● | ★ |  | ▲ |  |  |  |  |
| 11 |  |  |  | ▲ |  | ○ |  |  |
| 12 |  |  |  | ▲ |  | ○ |  |  |
| 13 | ● | ★ | ☆ | ▲ | △ | ○ | ● |  |
| 14 |  | ★ | ☆ | ▲ | △ |  |  |  |
| 15 |  | ★ | ☆ | ▲ | △ |  |  |  |
| 16 | ● | ★ |  | ▲ |  |  |  |  |
| 17 |  |  |  | ▲ |  | ○ |  |  |
| 18 |  |  |  | ▲ |  | ○ |  |  |
| 19 | ● | ★ | ☆ | ▲ | △ | ○ | ● |  |
| 20 |  | ★ | ☆ | ▲ | △ |  |  |  |
| 21 |  | ★ | ☆ | ▲ | △ |  |  |  |
| 22 | ● | ★ |  | ▲ |  |  |  |  |
| 23 |  |  |  | ▲ |  | ○ |  |  |
| 24 |  |  |  | ▲ |  | ○ |  |  |
| 25 | ● | ★ | ☆ | ▲ | △ | ○ | ● |  |
| 26 |  | ★ | ☆ | ▲ | △ |  |  |  |
| 27 |  | ★ | ☆ | ▲ | △ |  |  |  |
| 28 | ● | ★ | ☆ | ▲ | △ |  |  |  |
| 29 |  |  |  | ▲ |  |  |  |  |
| 30 |  |  |  | ▲ |  | ○ |  |  |
| 31 | ● | ★ | ☆ | ▲ | △ | ○ |  | **※** |

**Supplemental Table 4**. *In vitro* off-target pharmacological profile of cofrogliptin

| **Assay Name** | **Species** | **Concentration (μM)** | **Inhibition (%)** |
| --- | --- | --- | --- |
| ATPase, Na^+^/K^+^, Heart, Pig | pig | 10 | 2 |
| Cholinesterase, Acetyl, ACES | human | 10 | -8 |
| Cyclooxygenase COX-1 | human | 10 | 0 |
| Cyclooxygenase COX-2 | human | 10 | -11 |
| CYP450, 1A2 | human | 10 | 0 |
| CYP450, 2B6 | human | 10 | 19 |
| CYP450, 2C19 | human | 10 | 7 |
| CYP450, 2C8 | human | 10 | 7 |
| CYP450, 2C9 | human | 10 | 0 |
| CYP450, 2D6 | human | 10 | 9 |
| CYP450, 3A4 | human | 10 | 3 |
| Monoamine Oxidase MAO-A | human | 10 | 0 |
| Monoamine Oxidase MAO-B | human | 10 | 5 |
| Peptidase, Angiotensin Converting Enzyme | rabbit | 10 | 12 |
| Peptidase, CASP1 (Caspase 1) | human | 10 | 1 |
| Peptidase, CASP2 (Caspase 2) | human | 10 | -1 |
| Peptidase, CASP3 (Caspase 3) | human | 10 | -10 |
| Peptidase, CASP4 (Caspase 4) | human | 10 | 5 |
| Peptidase, CASP5 (Caspase 5) | human | 10 | 4 |
| Peptidase, CASP6 (Caspase 6) | human | 10 | 2 |
| Peptidase, CASP7 (Caspase 7) | human | 10 | -2 |
| Peptidase, CASP8 (Caspase 8) | human | 10 | 1 |
| Peptidase, CASP9 (Caspase 9) | human | 10 | 1 |
| Peptidase, CTSB (Cathepsin B) | human | 10 | -13 |
| Peptidase, CTSG (Cathepsin G) | human | 10 | 6 |
| Peptidase, CTSH (Cathepsin H) | human | 10 | -7 |
| Peptidase, Factor Xa | human | 10 | 4 |
| Peptidase, Thrombin | human | 10 | 1 |

**Supplemental Table 4**. *In vitro* off-target pharmacological profile of cofrogliptin (continuing)

| **Assay Name** | **Species** | **Concentration (μM)** | **Inhibition (%)** |
| --- | --- | --- | --- |
| Peptidase, Trypsin | human | 10 | 0 |
| Phosphodiesterase PDE3 | human | 10 | 7 |
| Phosphodiesterase PDE4 | human | 10 | 1 |
| Protein Serine/Threonine Kinase, PKC, Non-Selective | rat | 10 | 8 |
| Protein Tyrosine Kinase, Insulin Receptor | human | 10 | -9 |
| Protein Tyrosine Kinase, LCK | human | 10 | -4 |
| Adenosine A_1_ | human | 10 | 2 |
| Adenosine A_2A_ | human | 10 | -2 |
| Adenosine A_3_ | human | 10 | 2 |
| Adrenergic α_1A_ | rat | 10 | 3 |
| Adrenergic α_1B_ | rat | 10 | -2 |
| Adrenergic α_1D_ | human | 10 | 15 |
| Adrenergic α_2A_ | human | 10 | 9 |
| Adrenergic α_2B_ | human | 10 | 8 |
| Adrenergic β_1_ | human | 10 | -3 |
| Adrenergic β_2_ | human | 10 | 1 |
| Androgen (Testosterone) | human | 10 | 9 |
| Angiotensin AT_1_ | human | 10 | 1 |
| Bradykinin B_1_ | human | 10 | 11 |
| Bradykinin B_2_ | human | 10 | 0 |
| Calcium Channel L-Type, Benzothiazepine | rat | 10 | 13 |
| Calcium Channel L-Type, Dihydropyridine | rat | 10 | -4 |
| Calcium Channel L-Type, Phenylalkylamine | rat | 10 | 18 |
| Calcium Channel N-Type | rat | 10 | -1 |
| Cannabinoid CB_1_ | human | 10 | -2 |
| Cannabinoid CB_2_ | human | 10 | 1 |
| Chemokine CCR1 | human | 10 | -3 |

**Supplemental Table 4**. *In vitro* off-target pharmacological profile of cofrogliptin (continuing)

| **Assay Name** | **Species** | **Concentration (μM)** | **Inhibition (%)** |
| --- | --- | --- | --- |
| Chemokine CXCR2 (IL-8R_B_) | human | 10 | 15 |
| Cholecystokinin CCK_1_ (CCK_A_) | human | 10 | 5 |
| Cholecystokinin CCK_2_ (CCK_B_) | human | 10 | -2 |
| Dopamine D_1_ | human | 10 | 6 |
| Dopamine D_2L_ | human | 10 | 2 |
| Dopamine D_2S_ | human | 10 | 4 |
| Dopamine D_3_ | human | 10 | 4 |
| Dopamine D_4.2_ | human | 10 | 5 |
| Endothelin ET_A_ | human | 10 | -4 |
| Endothelin ET_B_ | human | 10 | 1 |
| Epidermal Growth Factor (EGF) | human | 10 | -5 |
| Estrogen ERα | human | 10 | 7 |
| GABA_A_, Chloride Channel, TBOB | rat | 10 | 0 |
| GABA_A_, Flunitrazepam, Central | rat | 10 | -7 |
| GABA_A_, Muscimol, Central | rat | 10 | -2 |
| GABA_A_, Ro-15-1788, Hippocampus | rat | 10 | 6 |
| GABA_B1A_ | human | 10 | -2 |
| Glucocorticoid | human | 10 | 6 |
| Glutamate, AMPA | rat | 10 | -13 |
| Glutamate, Kainate | rat | 10 | -2 |
| Glutamate, Metabotropic, mGlu_5_ | human | 10 | 5 |
| Glutamate, NMDA, Agonism | rat | 10 | 11 |
| Glutamate, NMDA, Glycine | rat | 10 | 15 |
| Glutamate, NMDA, Phencyclidine | rat | 10 | 3 |
| Glutamate, NMDA, Polyamine | rat | 10 | 21 |
| Glycine, Strychnine-Sensitive | rat | 10 | -12 |
| Histamine H_1_ | human | 10 | -10 |
| Histamine H_2_ | human | 10 | 6 |

**Supplemental Table 4**. *In vitro* off-target pharmacological profile of cofrogliptin (continuing)

| **Assay Name** | **Species** | **Concentration (μM)** | **Inhibition (%)** |
| --- | --- | --- | --- |
| Histamine H_3_ | human | 10 | 7 |
| Imidazoline I_2_, Central | rat | 10 | -24 |
| Interleukin IL-1 | mouse | 10 | 1 |
| Leukotriene, Cysteinyl CysLT_1_ | human | 10 | 2 |
| Melanocortin MC_1_ | human | 10 | -6 |
| Melanocortin MC_4_ | human | 10 | 6 |
| Melatonin MT_1_ | human | 10 | 7 |
| Muscarinic M_1_ | human | 10 | 13 |
| Muscarinic M_2_ | human | 10 | 5 |
| Muscarinic M_3_ | human | 10 | -1 |
| Muscarinic M_4_ | human | 10 | 11 |
| Neuropeptide Y Y_1_ | human | 10 | -8 |
| Neuropeptide Y Y_2_ | human | 10 | 2 |
| Nicotinic Acetylcholine | human | 10 | -9 |
| Nicotinic Acetylcholine α1, Bungarotoxin | human | 10 | -7 |
| Opiate δ_1_ (OP1, DOP) | human | 10 | 0 |
| Opiate κ (OP2, KOP) | human | 10 | 3 |
| Opiate μ (OP3, MOP) | human | 10 | 8 |
| Phorbol Ester | mouse | 10 | -3 |
| Platelet Activating Factor (PAF) | human | 10 | 18 |
| Potassium Channel [K_ATP_] | hamster | 10 | 1 |
| Potassium Channel hERG | human | 10 | -11 |
| PPARγ | human | 10 | 1 |
| Progesterone PR-B | human | 10 | 12 |
| Prostanoid EP_4_ | human | 10 | -9 |
| Purinergic P2X | rabbit | 10 | -8 |
| Purinergic P2Y | rat | 10 | -12 |
| Rolipram | rat | 10 | -4 |

**Supplemental Table 4**. *In vitro* off-target pharmacological profile of cofrogliptin (continuing)

| **Assay Name** | **Species** | **Concentration (μM)** | **Inhibition (%)** |
| --- | --- | --- | --- |
| Serotonin (5-Hydroxytryptamine) 5-HT_1A_ | human | 10 | 6 |
| Serotonin (5-Hydroxytryptamine) 5-HT_1B_ | human | 10 | 4 |
| Serotonin (5-Hydroxytryptamine) 5-HT_2A_ | human | 10 | -7 |
| Serotonin (5-Hydroxytryptamine) 5-HT_2B_ | human | 10 | 16 |
| Serotonin (5-Hydroxytryptamine) 5-HT_2C_ | human | 10 | 5 |
| Serotonin (5-Hydroxytryptamine) 5-HT_3_ | human | 10 | -2 |
| Sigma σ_1_ | human | 10 | 3 |
| Sodium Channel, Site 2 | rat | 10 | 22 |
| Tachykinin NK_1_ | human | 10 | 1 |
| Thyroid Hormone | rat | 10 | 3 |
| Transporter, Adenosine | guinea pig | 10 | 3 |
| Transporter, Dopamine (DAT) | human | 10 | -2 |
| Transporter, GABA | rat | 10 | -1 |
| Transporter, Norepinephrine (NET) | human | 10 | 3 |
| Transporter, Serotonin (5-Hydroxytryptamine) (SERT) | human | 10 | -3 |
| Vasopressin V_1A_ | human | 10 | -1 |

**Supplemental Table 5.** The percentage decrease in the area under blood glucose increment-time curve (AUC_Glu, incre_) in each group following glucose loading in ICR mice

| **Group** | **Percentage decrease of AUC_Glu, incre_ (%)** | | | |
| --- | --- | --- | --- | --- |
|  | **24 hours** | **48 hours** | **72 hours** | **96 hours** |
| MK3102, 10 mg/kg | 37.65 | 21.18 | 12.35 | 6.36 |
| Cofrogliptin, 1 mg/kg | 27.35 | 29.75 | 13.78 | 8.69 |
| Cofrogliptin, 3 mg/kg | 32.90 | 35.89 | 15.33 | 8.79 |
| Cofrogliptin, 10 mg/kg | 40.03 | 36.54 | 20.95 | 5.83 |

**Supplemental Table 6.** Effects of cofrogliptin on active GLP-1 levels in ICR mice

| **Group** | | **Active GLP-1 (pM)*** | **Percentage increase of active GLP-1 (%)** |
| --- | --- | --- | --- |
| Vehicle | | 3.79 ± 0.13 | -- |
| 24 hours | MK3102, 10 mg/kg | 7.66 ± 0.98 | 102.11 |
|  | Cofrogliptin, 1 mg/kg | 5.48 ± 0.47 | 44.59 |
|  | Cofrogliptin, 3 mg/kg | 7.97 ± 0.70 | 110.29 |
|  | Cofrogliptin, 10 mg/kg | 9.49 ± 1.03 | 150.40 |
| 48 hours | MK3102, 10 mg/kg | 4.60 ± 0.32 | 21.37 |
|  | Cofrogliptin, 1 mg/kg | 4.59 ± 0.21 | 21.11 |
|  | Cofrogliptin, 3 mg/kg | 5.14 ± 0.46 | 35.62 |
|  | Cofrogliptin, 10 mg/kg | 6.17 ± 0.48 | 62.80 |
| 72 hours | MK3102, 10 mg/kg | 4.39 ± 0.21 | 15.83 |
|  | Cofrogliptin, 1 mg/kg | 4.25 ± 0.14 | 12.14 |
|  | Cofrogliptin, 3 mg/kg | 4.98 ± 0.26 | 31.40 |
|  | Cofrogliptin, 10 mg/kg | 5.39 ± 0.21 | 42.22 |
| 96 hours | MK3102, 10 mg/kg | 4.18 ± 0.11 | 10.29 |
|  | Cofrogliptin, 1 mg/kg | 3.93 ± 0.07 | 3.69 |
|  | Cofrogliptin, 3 mg/kg | 4.00 ± 0.11 | 5.54 |
|  | Cofrogliptin, 10 mg/kg | 5.09 ± 1.11 | 34.30 |

Note: *Data are expressed as mean ± SEM. n=12 per group.

**Supplemental Table 7.** Effects of cofrogliptin on glycated hemoglobin (HbA1c), fructosamine (FRA), and serum insulin in *ob/ob* mice

| **Group** | **HbA1c (%) ^a^** | **FRA (μmoL/L)** | **Insulin (ng/mL)** |
| --- | --- | --- | --- |
| Wide-type | 3.67 ± 0.11 | 228.21 ± 3.88 | 0.29 ± 0.02 |
| Vehicle | 5.34 ± 0.24 | 348.39 ± 9.71 | 5.10 ± 0.42 |
| MK3102, 30 mg/kg | 4.61 ± 0.15 | 314.02 ± 16.30 | 10.85 ± 0.83 |
| Cofrogliptin, 1 mg/kg | 5.17 ± 0.22 | 327.93 ± 9.06 | 6.14 ± 0.67 |
| Cofrogliptin, 3 mg/kg | 4.32 ± 0.18 | 311.89 ± 13.10 | 7.22 ± 0.93 |
| Cofrogliptin, 10 mg/kg | 4.47 ± 0.27 | 300.48 ± 17.39 | 9.36 ± 1.07 |

Note: ^a^ Adapted from Zhang C., et al. (2020). Data are expressed as mean ± SEM. n=12 per group.

**Supplemental Table 8.** Effects of cofrogliptin on serum lipid metabolism-related indicators, total protein (TP), and albumin (ALB) in *ob/ob* mice

| **Group** | **TC**  **(mmoL/L)** | **TG**  **(mmoL/L)** | **HDL-C**  **(mmoL/L)** | **LDL-C (mmoL/L)** | **FFA**  **(mmoL/L)** | **TP**  **(g/L)** | **ALB**  **(g/L)** |
| --- | --- | --- | --- | --- | --- | --- | --- |
| Wide-type | 2.05 ± 0.08 | 0.44 ± 0.03 | 1.41 ± 0.06 | 0.26 ± 0.01 | 0.52 ± 0.03 | 48.77 ± 1.32 | 29.94 ± 0.58 |
| Vehicle | 5.77 ± 0.28 | 0.67 ± 0.09 | 3.84 ± 0.22 | 1.30 ± 0.10 | 1.19 ± 0.09 | 56.85 ± 1.75 | 32.18 ± 0.80 |
| MK3102,  30 mg/kg | 6.32 ± 0.30 | 0.62 ± 0.04 | 4.33 ± 0.17 | 1.38 ± 0.12 | 0.95 ± 0.07 | 64.33 ± 1.71 | 34.98 ± 0.57 |
| Cofrogliptin,  1 mg/kg | 5.50 ± 0.32 | 0.58 ± 0.10 | 3.83 ± 0.22 | 1.12 ± 0.10 | 0.94 ± 0.09 | 55.22 ± 1.93 | 31.57 ± 0.76 |
| Cofrogliptin,  3 mg/kg | 5.69 ± 0.38 | 0.60 ± 0.07 | 3.85 ± 0.22 | 1.28 ± 0.12 | 0.88 ± 0.06 | 57.27 ± 1.78 | 32.50 ± 0.75 |
| Cofrogliptin,  10 mg/kg | 5.82 ± 0.30 | 0.46 ± 0.02 | 4.01 ± 0.21 | 1.27 ± 0.10 | 0.82 ± 0.06 | 58.13 ± 1.89 | 32.68 ± 0.66 |

Note: Data are expressed as mean ± SEM. n=12 per group.

**Supplemental Table 9.** Effects of cofrogliptin on hepatic triglyceride content in *ob/ob* mice

| **Group** | **Hepatic triglyceride (HT, mg/g)** | **Prcentage increase of HT (%)** |
| --- | --- | --- |
| Wide-type | 10.85 ± 1.30 | -- |
| Vehicle | 133.36 ± 14.16 | -- |
| MK3102, 30 mg/kg | 126.56 ± 11.30 | 5.1 |
| Cofrogliptin, 1 mg/kg | 103.10 ± 10.88 | 22.7 |
| Cofrogliptin, 3 mg/kg | 94.65 ± 7.37 | 29.0 |
| Cofrogliptin, 10 mg/kg | 117.02 ± 9.67 | 12.2 |

Note: Data are expressed as mean ± SEM. n=12 per group.

**Supplemental Table 10.** Effects of cofrogliptin on adipose content in *ob/ob* mice

| **Group** | **Epididymal fat** | | **Mesenteric fat** | | **Scapular fat** | |
| --- | --- | --- | --- | --- | --- | --- |
|  | **Weight (g)** | **Tissue to body weight ratio** | **Weight (g)** | **Tissue to body weight ratio** | **Weight (g)** | **Tissue to body weight ratio** |
| Wide-type | 0.009 ± 0.001 | 0.041 ± 0.004 | 0.103 ± 0.0.18 | 0.481 ± 0.071 | 0.055 ± 0.005 | 0.266 ± 0.021 |
| Vehicle | 0.054 ± 0.003 | 0.100 ± 0.006 | 1.721 ± 0.046 | 3.274 ± 0.065 | 0.276 ± 0.026 | 0.525 ± 0.049 |
| MK3102,  30 mg/kg | 0.042 ± 0.004 | 0.077 ± 0.008 | 1.935 ± 0.052 | 3.467 ± 0.069 | 0.266 ± 0.021 | 0.478 ± 0.038 |
| Cofrogliptin,  1 mg/kg | 0.047 ± 0.004 | 0.084 ± 0.007 | 1.817 ± 0.109 | 3.363 ± 0.164 | 0.281 ± 0.020 | 0.529 ± 0.045 |
| Cofrogliptin,  3 mg/kg | 0.045 ± 0.007 | 0.087 ± 0.015 | 1.756 ± 0.086 | 3.336 ± 0.105 | 0.297 ± 0.022 | 0.566 ± 0.041 |
| Cofrogliptin,  10 mg/kg | 0.051 ± 0.003 | 0.095 ± 0.004 | 1.923 ± 0.062 | 3.479 ± 0.107 | 0.281 ± 0.025 | 0.508 ± 0.043 |

Note: Data are expressed as mean ± SEM. n=12 per group.

**Supplemental Table 10.** Effects of cofrogliptin on adipose content in *ob/ob* mice (continuing)

| **Group** | **Subcutaneous fat** | | **Perirenal fat** | | **Inguinal fat** | |
| --- | --- | --- | --- | --- | --- | --- |
|  | **Weight (g)** | **Tissue to body weight ratio** | **Weight (g)** | **Tissue to body weight ratio** | **Weight (g)** | **Tissue to body weight ratio** |
| Wide-type | 0.164 ± 0.015 | 0.793 ± 0.071 | 0.042 ± 0.006 | 0.197 ± 0.024 | 0.169 ± 0.014 | 0.813 ± 0.055 |
| Vehicle | 3.855 ± 0.247 | 7.352 ± 0.510 | 0.805 ± 0.077 | 1.556 ± 0.167 | 3.750 ± 0.177 | 7.102 ± 0.240 |
| MK3102,  30 mg/kg | 4.042 ± 0.246 | 7.261 ± 0.448 | 0.794 ± 0.069 | 1.426 ± 0.126 | 3.852 ± 0.143 | 6.895 ± 0.207 |
| Cofrogliptin,  1 mg/kg | 3.791 ± 0.246 | 7.113 ± 0.535 | 0.828 ± 0.076 | 1.539 ± 0.138 | 3.290 ± 0.207 | 6.135 ± 0.389 |
| Cofrogliptin,  3 mg/kg | 3.799 ± 0.212 | 7.309 ± 0.481 | 0.812 ± 0.094 | 1.567 ± 0.192 | 3.647 ± 0.139 | 6.978 ± 0.266 |
| Cofrogliptin,  10 mg/kg | 4.096 ± 0.297 | 7.400 ± 0.509 | 0.689 ± 0.052 | 1.249 ± 0.097 | 3.846 ± 0.218 | 6.920 ± 0.321 |

Note: Data are expressed as mean ± SEM. n=12 per group.

**Supplemental Table 11.** Effects of cofrogliptin on organ weight in *ob/ob* mice

| **Group** | **Liver** | | **Kidney** | | **Spleen** | |
| --- | --- | --- | --- | --- | --- | --- |
|  | **Weight (g)** | **Tissue to body weight ratio** | **Weight (g)** | **Tissue to body weight ratio** | **Weight (g)** | **Tissue to body weight ratio** |
| Wide-type | 0.906 ± 0.034 | 0.044 ± 0.001 | 0.250 ± 0.011 | 1.202 ± 0.024 | 0.133 ± 0.015 | 0.636 ± 0.062 |
| Vehicle | 3.289 ± 0.207 | 0.062 ± 0.003 | 0.391 ± 0.013 | 0.743 ± 0.022 | 0.136 ± 0.017 | 0.259 ± 0.032 |
| MK3102,  30 mg/kg | 3.409 ± 0.144 | 0.061 ± 0.003 | 0.383 ± 0.017 | 0.688 ± 0.031 | 0.203 ± 0.039 | 0.362 ± 0.069 |
| Cofrogliptin,  1 mg/kg | 3.159 ± 0.268 | 0.058 ± 0.004 | 0.369 ± 0.017 | 0.685 ± 0.023 | 0.154 ± 0.034 | 0.297 ± 0.072 |
| Cofrogliptin,  3 mg/kg | 3.086 ± 0.195 | 0.059 ± 0.003 | 0.381 ± 0.014 | 0.730 ± 0.029 | 0.206 ± 0.045 | 0.380 ± 0.072 |
| Cofrogliptin,  10 mg/kg | 3.404 ± 0.164 | 0.061 ± 0.002 | 0.383 ± 0.015 | 0.695 ± 0.029 | 0.122 ± 0.014 | 0.220 ± 0.026 |

Note: Data are expressed as mean ± SEM. n=12 per group.

**Supplemental Table 11.** Effects of cofrogliptin on organ weight in *ob/ob* mice (continuing)

| **Group** | **Pancreas** | | **Heart** | |
| --- | --- | --- | --- | --- |
|  | **Weight (g)** | **Tissue to body weight ratio** | **Weight (g)** | **Tissue to body weight ratio** |
| Wide-type | 0.194 ± 0.012 | 0.938 ± 0.056 | 0.116 ± 0.008 | 0.558 ± 0.035 |
| Vehicle | 0.269 ± 0.008 | 0.514 ± 0.021 | 0.131 ± 0.008 | 0.251 ± 0.017 |
| MK3102,  30 mg/kg | 0.280 ± 0.010 | 0.503 ± 0.017 | 0.148 ± 0.007 | 0.266 ± 0.013 |
| Cofrogliptin,  1 mg/kg | 0.306 ± 0.017 | 0.568 ± 0.029 | 0.120 ± 0.008 | 0.224 ± 0.013 |
| Cofrogliptin,  3 mg/kg | 0.265 ± 0.009 | 0.507 ± 0.017 | 0.132 ± 0.008 | 0.254 ± 0.016 |
| Cofrogliptin,  10 mg/kg | 0.259 ± 0.019 | 0.465 ± 0.031 | 0.140 ± 0.007 | 0.252 ± 0.011 |

Note: Data are expressed as mean ± SEM. n=12 per group.

**Supplemental Table 12.** PK parameters of cofrogliptin after single oral administration in SD rats

| **PK parameter** | **3 mg/kg** | | | **10 mg/kg** | | | **30 mg/kg** | | |
| --- | --- | --- | --- | --- | --- | --- | --- | --- | --- |
|  | **Male** | **Female** | **Total** | **Male** | **Female** | **Total** | **Male** | **Female** | **Total** |
| t_1/2_ (h) | 28 | 57 | 42 | 19 | 55 | 37 | 31 | 47 | 39 |
| t_max_ (h) | 0.5 | 2.7 | 1.6 | 1.3 | 3 | 2.2 | 2.3 | 3.3 | 2.8 |
| C_max_  (ng·mL^-1^) | 9460 | 10698 | 10079 | 25945 | 33139 | 29542 | 58647 | 71780 | 65214 |
| AUC_0-t_ (ng·h·mL^-1^) | 82579 | 401129 | 241854 | 310584 | 1336611 | 823598 | 608052 | 2355362 | 1481707 |

**Supplemental Table 13.** PK parameters of cofrogliptin after single oral administration in beagle dogs

| **PK parameter** | **1 mg/kg** | | | **3 mg/kg** | | | **10 mg/kg** | | |
| --- | --- | --- | --- | --- | --- | --- | --- | --- | --- |
|  | **Male** | **Female** | **Total** | **Male** | **Female** | **Total** | **Male** | **Female** | **Total** |
| t_1/2_ (h) | 21 | 22 | 21 | 19 | 19 | 19 | 18 | 16 | 17 |
| t_max_ (h) | 1.5 | 1.3 | 1.4 | 1 | 1.2 | 1.1 | 1.8 | 1.7 | 1.8 |
| C_max_  (ng·mL^-1^) | 1561 | 1945 | 1753 | 4582 | 4754 | 4668 | 12257 | 16032 | 14144 |
| AUC_0-t_ (ng·h·mL^-1^) | 22790 | 25433 | 24111 | 74140 | 64818 | 69479 | 233804 | 276730 | 255267 |

**Supplemental Table 14.** PK parameters of cofrogliptin and MK3102 after single oral administration in rhesus monkeys

| **PK parameter** | **Cofrogliptin** | **MK3102** |
| --- | --- | --- |
|  | **10 mg/kg** | **10 mg/kg** |
| t_1/2_ (h) | 64.9 | 53.9 |
| t_max_ (h) | 3.00 | 2.00 |
| C_max_ (ng·mL^-1^) | 4917 | 5900 |
| AUC_0-t_ (ng·h·mL^-1^) | 191114 | 145273 |

**Supplemental references**

CrystalChem. *The manufacturer's instructions for the ultra-sensitive mouse insulin ELISA kit [90082]*. Available at: <https://www.crystalchem.com/media/catalog/product/9/0/90082b.pdf>.

Mu J, Woods J, Zhou YP, et al. (2006) Chronic inhibition of dipeptidyl peptidase-4 with a sitagliptin analog preserves pancreatic beta-cell mass and function in a rodent model of type 2 diabetes. *Diabetes* 55: 1695-1704. <https://doi.org/10.2337/db05-1602>.
